# Controlled Measurement and Comparative Analysis of Cellular Components in *E*. *coli* Reveals Broad Regulatory Changes in Response to Glucose Starvation

**DOI:** 10.1371/journal.pcbi.1004400

**Published:** 2015-08-14

**Authors:** John R. Houser, Craig Barnhart, Daniel R. Boutz, Sean M. Carroll, Aurko Dasgupta, Joshua K. Michener, Brittany D. Needham, Ophelia Papoulas, Viswanadham Sridhara, Dariya K. Sydykova, Christopher J. Marx, M. Stephen Trent, Jeffrey E. Barrick, Edward M. Marcotte, Claus O. Wilke

**Affiliations:** 1 Institute for Cellular and Molecular Biology, The University of Texas at Austin, Austin, Texas, United States of America; 2 Center for Systems and Synthetic Biology, The University of Texas at Austin, Austin, Texas, United States of America; 3 Department of Organismic and Evolutionary Biology, Harvard University, Boston, Massachusetts, United States of America; 4 Center for Computational Biology and Bioinformatics, The University of Texas at Austin, Austin, Texas, United States of America; 5 Faculty of Arts and Sciences Center for Systems Biology, Harvard University, Boston, Massachusetts, United States of America; 6 Department of Biological Sciences, University of Idaho, Moscow, Idaho, United States of America; 7 Institute for Bioinformatics and Evolutionary Studies, University of Idaho, Moscow, Idaho, United States of America; 8 Department of Infectious Diseases, College of Veterinary Medicine, University of Georgia, Athens, Georgia, United States of America; 9 Department of Molecular Biosciences, The University of Texas at Austin, Austin, Texas, United States of America; 10 Department of Integrative Biology, The University of Texas at Austin, Austin, Texas, United States of America; Hellas, Greece

## Abstract

How do bacteria regulate their cellular physiology in response to starvation? Here, we present a detailed characterization of *Escherichia coli* growth and starvation over a time-course lasting two weeks. We have measured multiple cellular components, including RNA and proteins at deep genomic coverage, as well as lipid modifications and flux through central metabolism. Our study focuses on the physiological response of *E*. *coli* in stationary phase as a result of being starved for glucose, not on the genetic adaptation of *E*. *coli* to utilize alternative nutrients. In our analysis, we have taken advantage of the temporal correlations within and among RNA and protein abundances to identify systematic trends in gene regulation. Specifically, we have developed a general computational strategy for classifying expression-profile time courses into distinct categories in an unbiased manner. We have also developed, from dynamic models of gene expression, a framework to characterize protein degradation patterns based on the observed temporal relationships between mRNA and protein abundances. By comparing and contrasting our transcriptomic and proteomic data, we have identified several broad physiological trends in the *E*. *coli* starvation response. Strikingly, mRNAs are widely down-regulated in response to glucose starvation, presumably as a strategy for reducing new protein synthesis. By contrast, protein abundances display more varied responses. The abundances of many proteins involved in energy-intensive processes mirror the corresponding mRNA profiles while proteins involved in nutrient metabolism remain abundant even though their corresponding mRNAs are down-regulated.

## Author Summary

Bacteria frequently experience starvation conditions in their natural environments. Yet how they modify their physiology in response to these conditions remains poorly understood. Here, we performed a detailed, two-week starvation experiment in *E*. *coli*. We exhaustively monitored changes in cellular components, such as RNA and protein abundances, over time. We subsequently compared and contrasted these measurements using novel computational approaches we developed specifically for analyzing gene-expression time-course data. Using these approaches, we could identify systematic trends in the *E*. *coli* starvation response. In particular, we found that cells systematically limit mRNA and protein production, degrade proteins involved in energy-intensive processes, and maintain or increase the amount of proteins involved in energy production. Thus, the bacteria assume a cellular state in which their ongoing energy use is limited while they are poised to take advantage of any nutrients that may become available.

## Introduction

Many global changes in cellular physiology occur during the growth of a typical laboratory culture of a microorganism, such as *Escherichia coli*, as it transitions from exponential growth to starvation where it eventually ceases dividing as nutrients become exhausted [1]. However, how these changes affect specific cellular components and processes is not fully known. Existing surveys, even if conducted at the genome scale, tend to have limited completeness, in at least two ways. First, most studies collect only one type of genome-scale data. For example, they either measure changes in gene expression, through RNA or protein levels, or they measure changes in metabolites. Second, technological limitations often prevent the detection of some subset of molecules in a category of interest. For example, small bacterial RNAs with key roles in regulation may be lost from a sample when using typical methods to purify “total” RNA from cells [2]. Furthermore, DNA microarray-based methods for profiling gene expression can only detect specific RNA sequences depending on the design of their probes, whereas RNA-seq transcriptomic methods theoretically recover all RNA species in a sample [3]. Similarly, in proteomics, 2-D gel electrophoresis approaches typically detect many fewer proteins than newer mass spectrometry based shotgun methods [4,5].

Moreover, while the short-term changes in cellular physiology that occur in a laboratory culture of *E*. *coli* have been the subject of intensive study, considerably less is known about the changes in cellular composition that occur during the long-term survival of *E*. *coli* and other non-spore-forming microbes under starvation, despite the likely prevalence of this condition in nature [6]. Most studies of this metabolic state have concentrated on the long-term survival of cells in rich medium [7]. Under these conditions, *E*. *coli* experience an ecological catastrophe in which 90–99% of the cells die within a few days due to pH and nutrient changes in the medium, and mutants emerge that continue to divide on the resources released from dead cells [8–10] Thus, these are studies of genetic adaptation to changed conditions rather than purely of changes in cellular physiology in stressed and starving, but genetically wild-type, cells.

Finally, most genome-wide analyses of gene regulation focus on comparing differential changes across only two or three distinct environmental conditions or between two different time points. These studies reveal a snapshot of global physiological regulation but they do not provide insight into the underlying dynamics of regulation. By studying the dynamics of gene regulation over time, we can develop an understanding of how a cell’s physiology is regulated in the face of a natural environment that may undergo frequent changes.

Here we performed a time course experiment of *E*. *coli* B REL606 growth and starvation up to two weeks. We used a chemically defined glucose-limited medium in which cells entered a starvation state but did not lose viability for at least one week. We collected genome-wide RNA and protein levels at multiple time points, as well as lipid-modification and central metabolic-flux data, all under identical, controlled experimental conditions. The resultant data set serves as a rich resource for computational models that span and integrate cellular sub-systems and for cataloguing and correlating the responses of specific genes and/or molecules across cellular subsystems during growth and long-term starvation. We analyzed these data using a novel, general approach for unbiased classification of expression time courses. We found that the mRNA pool was drastically reduced during starvation, possibly to limit new protein synthesis overall, and that some proteins declined rapidly in abundance, in proportion to their mRNAs, while others were buffered to rapid changes in their transcripts. Overall, we observed a pattern where starving *E*. *coli* cells employ transcriptional and translational/post-translational regulation to limit energy requirements while remaining capable of nutrient uptake and metabolism.

## Results

### Controlled measurements of multiple cellular components yield highly reproducible data and unprecedented depth of coverage

**Fig 1:**
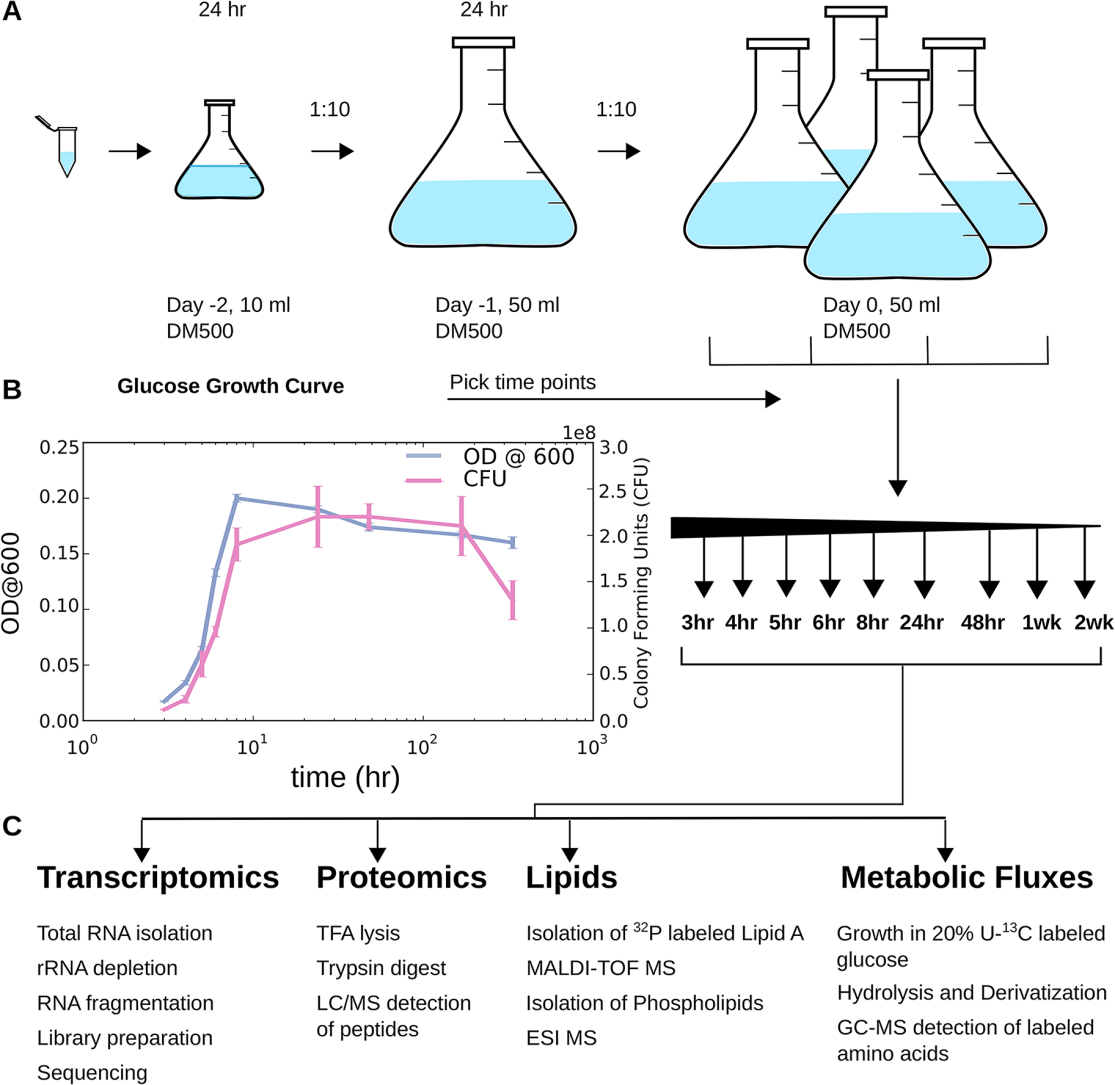
Overview of experimental design. Measurements of RNA, protein, lipids, and metabolic flux were taken under uniform growth and environmental conditions. (A) Long-term stationary phase experiment. The *E*. *coli* B REL606 strain was taken from a freezer stock and revived (day –2), diluted and regrown to precondition it to culture conditions for 24 h (day –1), and diluted then into several individual cultures to initiate the experiment. (B) The OD_600_ (blue curve) was measured to assess growth and optimal collection of time points. Nine time points were selected for this experiment, spanning three hours to two weeks. Cell viability was accessed at each time point by determining the number of colony forming units (CFU, purple curve). (C) For each sample an aliquot was removed from the culture for each experiment to be done, spun down, flash frozen, and used to measure RNA via RNA-seq, protein via LC/MS, lipids via MALDI-TOF MS and ESI MS, and metabolic flux via GC-MS. Metabolic flux samples were grown separately under identical conditions excepting the labeled U-^13^C glucose. Raw RNA and protein counts, calculated flux ratios, raw phospholipid MS peaks, and lipid A peaks for all time points are available in S1 File.

We grew multiple cultures of *E*. *coli* REL606, from the same stock, under identical growth conditions of long-term glucose starvation, in the same medium. The samples were subsequently distributed to different laboratories that measured RNA, protein, lipids, and central metabolic flux ratios. Freezer stocks of the REL606 strain were revived for 24 h, diluted and preconditioned for another 24 h, and diluted again to initiate the experimental time course (Fig 1A). Each biological replicate was performed on separate days. In a pilot experiment a growth curve was measured to determine informative time points for analysis (Fig 1B). Time points spanning three hours to two weeks were collected and used to measure RNA via RNA-seq, proteins via LC/MS, lipids via MALDI-TOF MS and ESI MS, and central metabolic fluxes via ^13^C labeled glucose and GC-MS (Fig 1C). In our conditions, the optical density at 600 nm (OD_600_) changed little once cells entered stationary phase (Fig 1B). Additionally, cell viability remained constant after entry to stationary phase at 24 h for up to one week. From one to two weeks, the number of viable cells per culture count decreased by 38% (Fig 1B).

We first assessed reproducibility of protein and RNA measurements. For both, we found that measurements from separate biological replicates correlated highly with each other. We saw Spearman correlations of 0.92, 0.92, and 0.95 between biological repeats of raw proteomics counts and correlations of 0.93, 0.93, and 0.94 for raw RNA-seq counts between the 3 h biological replicates (S1 Fig). Furthermore, we also compared the overlap in protein IDs between the first three time points (3, 4, and 5 hrs), when the cells were exponentially dividing and the protein concentrations were more-or-less at steady state, and we found a high overlap among these time points. Each single time point yielded just over 2600 protein IDs, any pair yielded just over 2300 common protein IDs, and all three time points yield over 2100 overlapping protein IDs (S2 Fig). Thus, our measurements were highly reproducible.

We next compared how many different RNA and protein species we detected compared to previous 'multi-omic' studies (Table 1). Yoon et al. used 2D gels and microarrays to measure 60 significantly changing proteins and 4,144 mRNAs in *E*. *coli* REL606, the same strain used in this study [11]. By comparison, at 3–4 h, we observed over 2,600 proteins, with ~1,200 that changed significantly at some point in the time course, along with 4,116 mRNAs, 85 tRNAs, and 89 other noncoding RNAs (ncRNAs), a category that is largely made up of small RNAs. Even though the total number of proteins Yoon et al. observed at early exponential phase was not reported [11], it was likely an order of magnitude less than our observations, if it followed the same pattern as the proteins found to have significant changes in expression. Taniguchi et al. measured protein and mRNA content of single cells using YFP fusions and FISH, resulting in the measurement of 1,018 proteins and 137 transcripts in an *E*. *coli* K12 strain [12]. Lewis et al. also measured ~1,000 proteins and RNA expression of 4,428 genes. Although these data sets were published separately, they were performed in the same lab and under similar conditions and thus were also comparable to a degree [13,14]. In summary, our proteomics measurements were far more complete than comparable studies, providing more than 1,000 additional protein observations than the most comprehensive other study, as many mRNAs as other studies, and additional data on tRNAs and ncRNAs.

**Table 1:** Comparison of data set completeness.

Publication	# Proteins	Cutoff	Technique	# RNAs	Cutoff	Technique
This study	2648* (at 3 h)	FDR <1%	Shotgun MS	4116 mRNA, 89 ncRNA, 85 tRNA (at 3 h)	Alignment quality >10	RNA-seq
[11]	60	fold change >2	2-D gel	4144 gene probes (REL606 strain)	NA	Microarray
[12]	1018	NA	YFP fusion	137	95% confidence	FISH
[13]	~1,000	P-value <0.05	Shotgun MS	-		
[14]				4428 gene probes	NA	Microarray
[4]	2053*	FDR <1%	SILAC	-		
[5]	2200	FDR <1%	FASP	-		
[15]				4161 mRNA, 133 ncRNA	%region mapped ≥50%	RNA-seq

*We counted proteins as observed if they appeared in at least 1 of 3 biological repeats, whereas Ref. [4] counted proteins that appeared in at least 1 of 2 biological repeats.

Our experiments also provided coverage comparable to or better than other experiments that focus on proteomics or RNA measurements alone. Using stable isotope labeling of amino acids (SILAC), Soares et al. observed 2,053 proteins in at least 1 of 2 biological repeats, at a false discovery rate (FDR) of <1% [4]. We measured 2,658 proteins in at least 1 of 3 biological repeats with around 2,200 protein IDs per sample using the same FDR cutoff. A more recent study, using the filter-aided sample preparation (FASP) method, also observed around 2,200 proteins per sample, comparable to our recovery [5]. Additionally, using RNA-seq, we recovered as many mRNAs as microarray approaches do, with the added benefit of measuring 89 ncRNAs and 85 tRNAs from the same sample. As a point of reference, previous RNA-seq experiments on the *E*. *coli* K-12 strain identified 133 putative ncRNAs and 4,161 mRNAs [15]. Thus our recovery of both proteins and RNA represents the state of the art of the field, far outperforming recent comparative studies. As an added benefit of our study, we also simultaneously characterized lipid A and phospholipid composition in cell membranes and measured flux ratios in central metabolism, covering a wider range of cellular components than previous comparison studies.

### Measured mRNAs are regulated in a comparatively more uniform manner compared to proteins

We next investigated changes in relative mRNA and protein abundance over time. Due to translational and post-translational regulation we expected differences in the response of mRNA transcripts and proteins after entry to stationary phase. mRNA counts at each time point were normalized via DESeq [16], relative to the total pool of mRNA, tRNA, and ncRNA. Protein counts at each time point were normalized relative to the total protein count.

**Fig 2:**
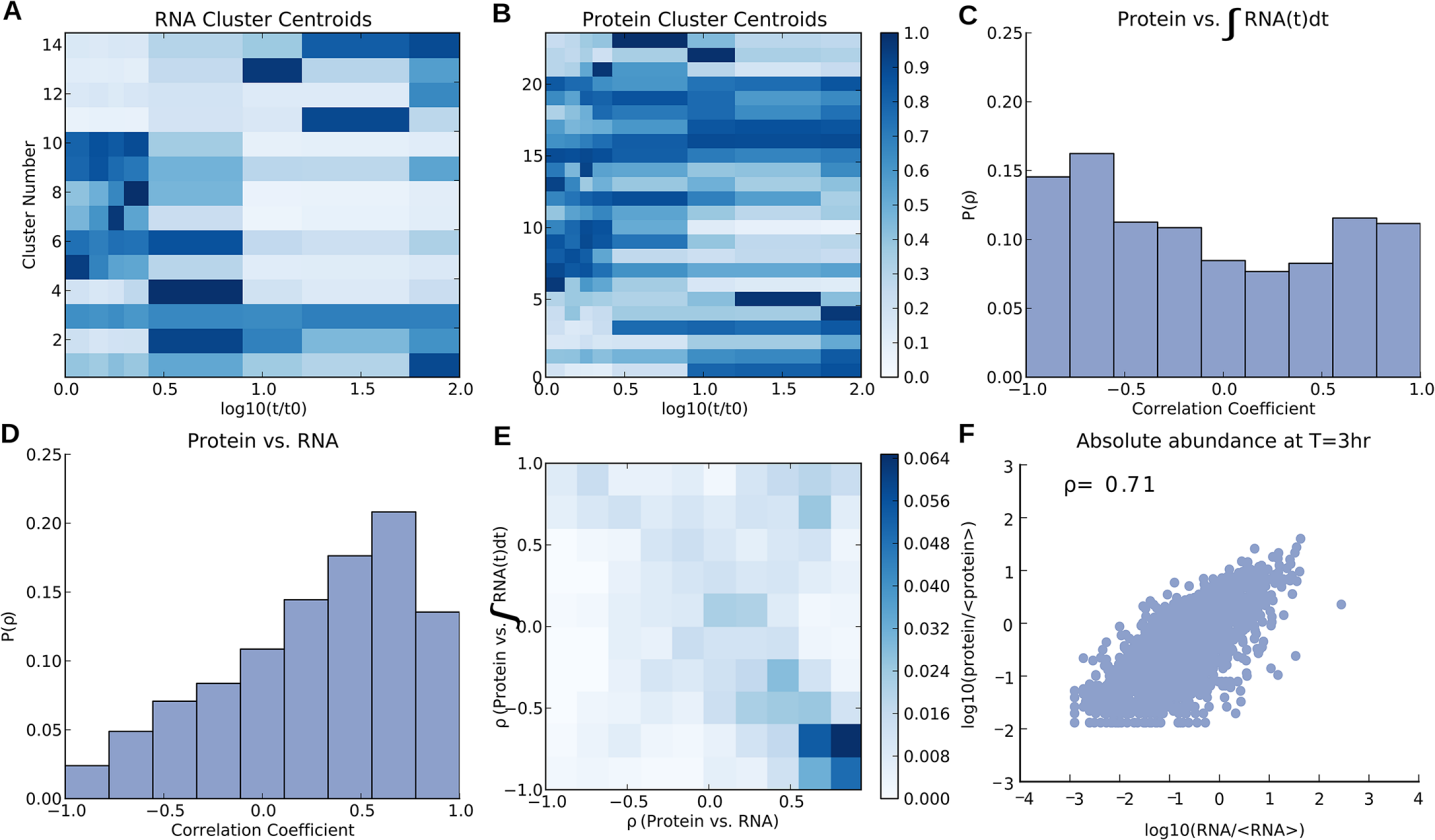
*K*-means clustering of mRNA and protein profiles in long-term stationary phase revealed trends in transcriptional and post-transcriptional regulation. mRNA and protein profiles, normalized to each molecule’s maximum value, were clustered by geometric distance using *K*-means clustering with 15 and 25 clusters, respectively. The cluster centroids were then plotted as heat maps with darker blue representing higher RNA or protein levels. (A) mRNA levels were largely shut off upon entry to stationary phase with some mRNA being transiently up-regulated during the transition between exponential and stationary growth. (B) Protein levels showed a much wider range of behaviors with some being up- or down-regulated for the duration of the experiment as well as for a short period of time during the transition from exponential to stationary growth. (C) Histogram of the correlation coefficients between individual protein levels and the time integral of mRNA expression. This model tests the limit of slow protein degradation, where protein levels are proportional to the cumulative sum of their respective transcripts. Approximately 15% of the protein levels integrated their transcript’s response over the entire duration of the experiment (with ρ>0.70). (D) Histogram of the correlation coefficient between relative protein levels and their corresponding (relative) transcripts. This model tests to what extent protein levels are proportional to their respective mRNA levels. Approximately 20% of the protein levels were proportional to their transcript’s response over the entire duration of the experiment (with ρ>0.70). (E) 2-D histogram of the correlation coefficient of protein vs. mRNA (on the *x*-axis) and the protein vs. the time integral of mRNA (*y*-axis). Darker colors indicate more genes in the given bin. We observed a strong anti-correlation between the two measures of dynamic correlation, indicating that these two quantities were largely mutually exclusive. (F) The correlation between all mRNA and protein levels for a single time point was strongest at 3 h (Spearman correlation coefficient ~0.71). Normalized mRNA and protein levels, both relative and absolute, used to generate the above figure are provided in S2 File.

To visualize changing mRNA and protein levels we compared and contrasted the general trends in the response of mRNA and proteins by way of *K*-means clustering. To simplify the analysis we focused on only those mRNAs and proteins that were changing significantly (as measured by false discovery rate and fold-change cutoff, respectively) throughout the time course, yielding a total of ~1900 significantly changing transcripts/proteins. To perform *K*-means clustering, an arbitrary choice for the number of clusters must be made such that the profiles are well separated into groups with unique and distinct behaviors. (We also developed an alternative classification approach that does not depend on such an arbitrary choice, see below.) We varied the number of clusters for both mRNA and protein profiles, and we found the best clustering performance, assessed by visual inspection, to be around 15 clusters for the mRNA profiles and 25 clusters for the protein profiles. Thus, the mRNAs appeared to respond in a more uniform manner than the proteins did. This finding is illustrated by the heat map of the cluster centroids of mRNA and protein (Fig 2A and 2B, respectively). The vast majority of the differentially regulated mRNAs were down-regulated, while the protein response was much less uniform. Additionally, the mRNA profiles showed a clear separation between early and late time points with a transition period around 6–8 h. After this transitional period of entry to a starved state, the transcription profiles remained relatively constant, with only minor changes in expression. At two weeks some of the transcripts began changing again, perhaps signaling a further shift in cell state.

As the cells ran out of glucose, overall demand for new protein synthesis was significantly decreased, demand for certain stress response proteins increased, and resources became limiting. New protein synthesis could be globally limited in at least three ways: by reducing the amounts of rRNAs, charged tRNAs, or mRNAs. To understand how these different RNA pools changed relative to each other, we calculated the relative amount of mRNA, tRNA, ncRNA, and rRNA present in both ribosome depleted and non-ribosome depleted samples (S3 Fig). In the non-ribosomal depleted case the fraction of rRNA changed very little throughout the course of the experiment while the tRNA fraction increased and the mRNA fraction decreased. In the ribosome depleted samples (in which we removed residual rRNA counts due to incomplete depletion before analysis), the tRNA fraction also increased as the mRNA fraction decreased, confirming that this effect was not due to sensitivity or sampling-bias issues resulting from rRNA dominating the RNA pool in the non-ribosome depleted sample.

We would like to emphasize that the above clustering of the RNA and protein abundances were performed independently of each other. Therefore, we could not directly compare individual clusters between Fig 2A and 2B. The next section addresses the correlation between absolute and relative changes in abundance of individual proteins and their transcripts.

### Differences in post-transcriptional regulation lead to differences in correlation between individual mRNA and protein time courses

While it has been observed that absolute levels of proteins do not necessarily correlate strongly with their corresponding transcripts, we expected at least a moderate correlation between absolute mRNA and protein levels at a given time point. We also expected a correlation within individual time courses between the relative levels of a protein and its transcript. To relate the relative levels of a protein to its transcript we had to account for the underlying dynamics of the time courses. We considered two limiting cases: At one extreme we assumed each protein had a degradation rate slower than the time scale of the experiment. At the other extreme we assumed each protein was degraded on a time scale that was fast compared to the time scale of the experiment. In the first limiting case proteins integrate their transcript levels over time. In the second limiting case (relative) protein levels track with their (relative) transcript level. Obviously, we expect that some proteins do not match either of these extreme cases but fall into an intermediate regime between the two.

Plotted in Fig 2C and 2D are histograms of the Spearman correlation coefficients (ρ) calculated for proteins vs. the integrals of their transcripts (integral regulation) and proteins vs. their transcripts (proportional regulation), respectively. Approximately 15% of the proteins correlated highly (ρ>0.70) with the integrals of their transcripts whereas approximately 20% correlated highly with their transcript levels. There was little overlap between the two sets, as can be seen by the strong anti-correlation in the 2D histogram in Fig 2E of protein versus the integral and proportional levels of mRNA. Genes that were proportionally regulated were enriched for, among other things, locomotion and cell division. Genes that were integrally regulated were enriched for glycerol, alditol, and polyol metabolism. For a full list of proteins that were either proportionally or integrally related to their transcripts see S1 and S2 Tables, respectively. Approximately 65% of proteins did not fit one of these limiting models of how transcript and protein abundance were correlated; they may experience intermediate protein degradation rates or their expression and activity may be controlled by more complex post-translational modifications.

To put proteins and RNA within a given sample on comparable absolute scales, we normalized protein counts using the APEX method [17] for absolute quantification, and we normalized mRNA counts to the length of each transcript. Both protein and mRNA levels were then averaged across all three biological replicates. Additionally, all proteins and mRNAs were scaled by the average of all proteins and mRNA.

The strongest absolute correlation, across the time course, between mRNA and protein occurred at three hours (Fig 2F, Spearman ρ = 0.71, *P* = 10^−224^). Absolute correlation between proteins and their corresponding transcripts were relatively strong for time points ≤8 h, with a correlation coefficient of ~0.71. After 8 h, when cells had entered a starved state, the correlation was much weaker, with correlations around 0.3–0.4 (S4 Fig). The correlation at three hours was somewhat higher than is usually observed for correlations between RNA and protein for other measured prokaryotes and eukaryotes, which typically have Spearman correlations around 0.5 between proteins and their transcripts [18–23].

### RNAs within an operon correlated strongly while proteins within an operon did not necessarily correlate strongly with each other

Genes within an operon are co-transcribed as a single RNA and thus are likely to be under the same transcriptional control. Differences in translational efficiency between genes often lead to larger differences in protein expression in the same operon, as regulation via changes in subcellular localization, post-translational modifications, or control of degradation rates may differently impact the activities of each of these proteins [24–27]. We expected to see a high correlation between counts of RNAs for each gene within an operon, as the genes within an operon are under the same transcriptional control; however, we expected there to be less correlation between proteins within an operon, as the proteins are not guaranteed to be subject to the same translational/post-translational regulation.

**Fig 3:**
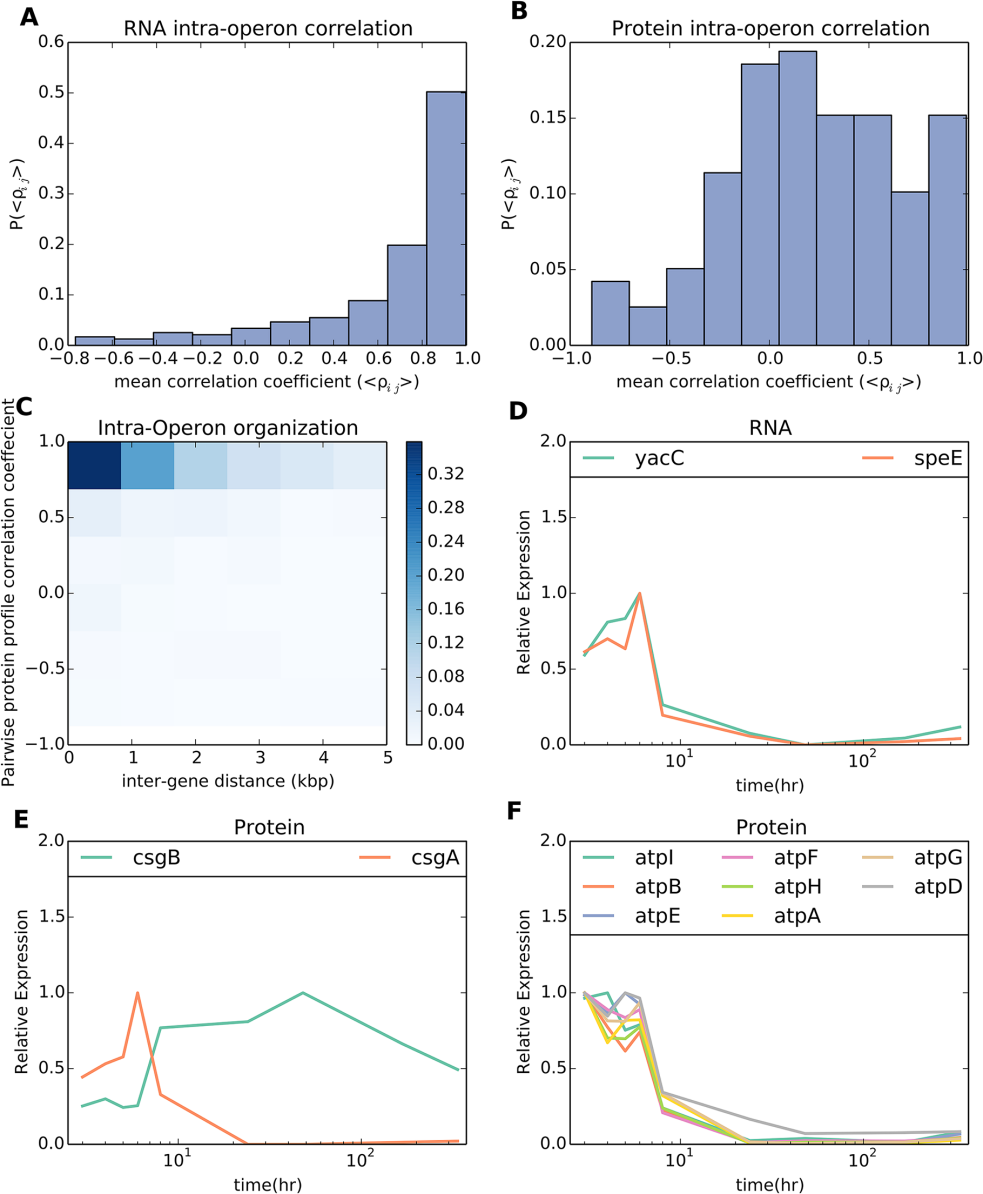
mRNA levels within an operon correlated strongly whereas protein levels generally did not. (A, B) Histograms of the median pairwise correlation coefficient between all possible pairs of mRNA and protein profiles, respectively, within an operon. (C) 2D Histogram of the pairwise correlation between proteins in the same operon (*y*-axis) and the inter-gene distance between the protein coding regions (*x*-axis). Darker colors represent higher correlation. Proteins that had a smaller inter-gene distance were more likely to have correlated profiles. (D) Example of mRNAs in the same operon that were highly correlated. (E, F) Examples of proteins in the same operon that were poorly and highly correlated, respectively.

As a measure of correlation of gene expression within an operon we took the average of the pairwise Spearman correlation coefficient for all possible pairs of transcripts and proteins within an operon. Approximately eighty percent of transcripts had a mean pairwise correlation coefficient greater than 0.8 within an operon (Fig 3A). On the other hand, less than fourteen percent of proteins had a mean pairwise correlation coefficient greater than 0.8 within an operon (Fig 3B). Genes closer together within an operon were more likely to have correlated protein profiles (see Fig 3C), which we took as evidence that distance between genes was a strong indicator of translational regulation. Also shown are a few examples of highly correlated transcripts and proteins for individual operons (Fig 3D, 3E and 3F, respectively).

### Energy-intensive processes are transcriptionally down-regulated while stress-related proteins are up-regulated upon entry to stationary phase

Typical analysis of RNA expression data often involves performing a hierarchical clustering of profiles followed by a term enrichment of subsets of genes found in the emerging patterns. In this approach the patterning that comes from hierarchical clustering can be arbitrary, depending on the level of the hierarchy one chooses to focus on. Here, instead, we sought to sort the time courses into general behaviors in an unbiased manner. To accomplish this goal we fit each individual mRNA and protein to a piecewise continuous curve (S5A Fig). This curve was defined by four free time parameters and three free amplitude parameters. To fit the curve we used a population-based differential evolution (DE) algorithm with the fitness function used in minimization scaled to the experimental error (see Methods). Thus, our algorithm provided confidence intervals for our fit based upon the variability in biological replicates.

To demonstrate the effectiveness of our fitting strategy we randomly selected five mRNA profiles and their respective fits (S5B–S5E Fig). Green circles show the average of three biological replicates with their standard deviations (green bars) and the blue line and bar show the average and standard deviation of the population of fits, respectively. Both the data and fit were normalized to the average of the time course. We also plotted histograms of the time scale parameters we found by fitting the piecewise continuous curve to our data (S6 Fig). The most informative time scales were *t*_1_, the time to first inflection, and *t*_2_+*t*_3_+*t*_4_, the time it takes for the profile to stop changing. The majority of proteins and their transcripts began changing before the 10 h mark (or just after the cells enter a starved state). Once the profiles began to change it took >10 h before they stopped changing again. However, in this case the apparent long time scale of proteins and transcripts changing could be due to the low time resolution of our experiment after the cells had entered a starved state.

**Fig 4:**
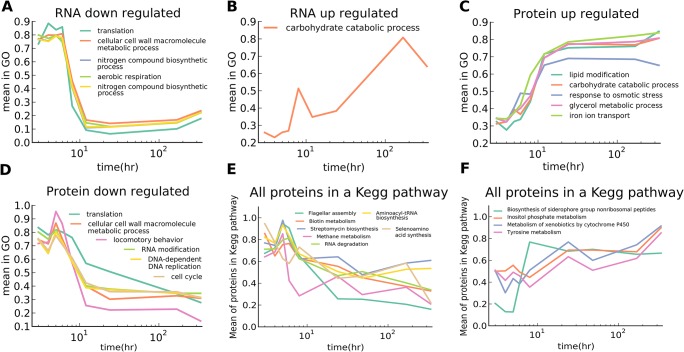
Flagellar genes and other energy intensive processes were down-regulated while stress-response genes were up-regulated. Fitting the mRNA and protein profiles allowed us to estimate the underlying dynamics and differential regulation of each gene, sorting them into high confidence categories describing their behavior. Genes were put into categories based upon whether they were up-regulated, down-regulated, transiently up-regulated, or transiently down-regulated. The mRNA or proteins in each category were then tested for enrichment of GO terms. (A, B) The average of the mRNAs in a given enriched GO term that were down- and up-regulated, respectively. (C, D) The average of the proteins in a given enriched term GO term that were down- and up-regulated, respectively. Amine biosynthesis was also enriched for mRNAs that were transiently up-regulated (not plotted) however no other terms for either mRNA or protein were enriched for the transiently up- or down-regulated categories. All functional clustering of GO enrichment terms for all categories, for both protein and mRNA, are provided in S3 and S4 Files, respectively. As a complementary approach we took the average of all proteins in a given pathway. (E, F) The average protein levels in the KEGG pathway, for KEGG pathways that changed significantly. All the other terms showed no significant change.

As can be seen in S5B–S5E Fig, there was generally good agreement between the data and model for mRNAs. Thus, the fits gave us reasonable estimates of the distribution of time scales involved in the response. S5F Fig shows the distribution of *t*_1_, the time to first inflection. Most of the mRNAs responded between 3–8 h, with a strong peak at around 6 h (when cells began entry to a starved state). To better understand the regulation of cellular processes (and mRNAs) in our dataset, we sorted the mRNA profiles into five general categories, defined on the basis of our fitted parameters: up-regulated, down-regulated, transiently up-regulated, transiently down-regulated, or ambiguous. The confidence intervals for our fits allowed sorting individual mRNAs into these five categories with high confidence. The mRNAs in the categories down-regulated and up-regulated showed significant enrichment for GO terms. The average of the mRNAs in each of these terms is shown in Fig 4A and 4B. Terms enriched in the set of down-regulated transcripts represented translation, carboxylic acid biosynthetic process, and nitrogen compound biosynthetic process. These processes were likely down-regulated for energy conservation purposes in the face of limiting resources. Terms enriched in the set of up-regulated transcripts represented carbohydrate catabolic processes.

To characterize the protein response we followed the same general strategy of fitting, classification, and GO enrichment as we had done for the RNA profiles. The distribution of the time to first inflection for the proteins was a little broader than for the mRNAs. However, the first-inflection times still mostly fell into the range of 3–8 h, and very few proteins had not responded by the time the cells entered a starved state. There were many proteins that were present for the duration of the time course, compared to the mRNAs where very few remained present for the entire duration of the experiment. Fig 4C shows the average abundance of the proteins in a given GO term that were enriched in the set of proteins that were being up-regulated. As in the case of down-regulated RNAs these proteins were likely down-regulated to conserve energy, and they included proteins involved in translation and locomotion. Up-regulated proteins were, like the up-regulated transcripts, involved in carbohydrate catabolism but also included terms involved in stress response and metabolism of glycerol. The average protein abundances for GO terms being down-regulated had a much wider distribution of decay times compared to the RNAs being down-regulated, likely due to differing protein degradation rates (and/or thermodynamic stability) (Fig 4D).

As a complementary approach we also averaged all proteins in a given KEGG pathway regardless of their behavior. Many pathways showed little to no differential regulation, on average, in their protein levels. Pathways that changed cohesively are plotted in Fig 4E and 4F, depending on whether they were down- or up-regulated, respectively. As in the previous term-enrichment analysis, we saw motility to be down-regulated, as well as other energy consuming processes involved in metabolism and biosynthesis. Interestingly, biosynthesis of siderophores was up-regulated, likely due to do increased demands for or reduced supply of iron.

### Central metabolic fluxes were consistent during exponential growth

We used flux ratio analysis to measure the relative metabolic fluxes passing through different branches of central metabolism [28,29]. To measure flux ratios we used the FiatFlux software that fits a metabolic model to the amino acid labeling pattern [30]. Importantly, this analysis represents the integral of metabolism until the time at which the measurement was taken. As there was little *ab-initio* protein synthesis after the cells stopped growing (after ~8 h), we did not include the flux ratios after this point, except for the two-week time point. Our major observation was that there was little change in flux ratios throughout growth, and for most of the experiment this initial labeling remained (S7A–S7I Fig). Interestingly, we observed changes at two weeks in the flux ratio in P5P from G6P lower branch (S7G Fig). Given that there is not expected to be any net synthesis of amino acids after growth ceased, we cannot use the steady-state approach to interpret these data. They do suggest, however, that either internal amino acid recycling or some *de novo* amino acid synthesis from recycling nutrients released by dead cells occurred after one week.

### Lipids are modified in stationary phase for up to two weeks

**Fig 5:**
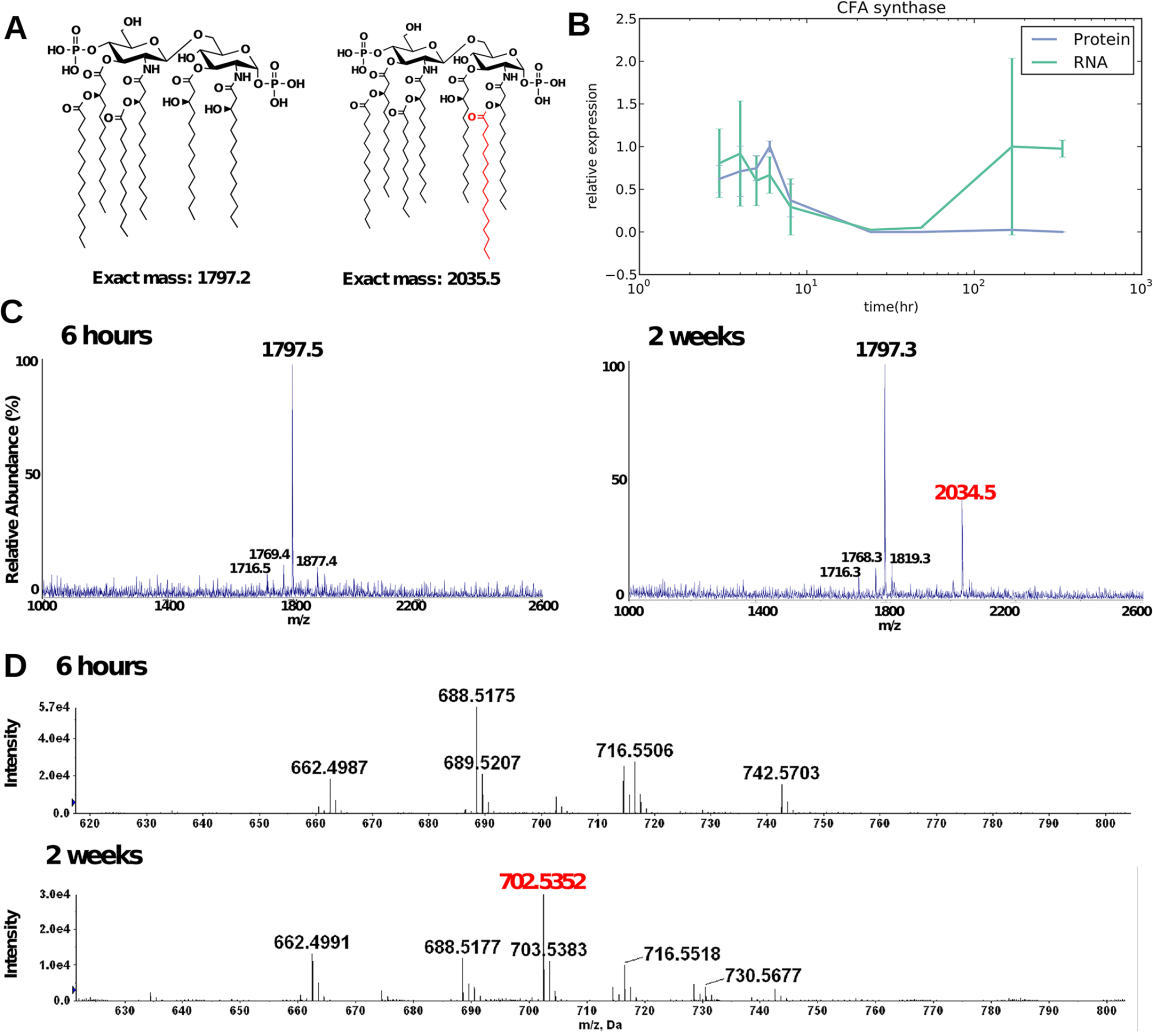
Lipid A and phospholipids were modified starting at 6 hours and these modifications continued to accumulate for two weeks. Lipid A and phospholipids were extracted from all samples for analysis by negative ion MALDI-TOF and ESI-MS mass spectrometry, respectively, and the 6 h and two week representative samples are shown in this figure. (A) Activation of the acyl-transferase PagP adds a C_16_ chain to lipid A on the 2-position primary acyl chain, resulting in a m/z of ~2035. (B) Modification of phospholipids by cyclopropanation of one unsaturated double bond is catalyzed by CFA synthase. Transcripts of CFA synthase increased at late times (green) consistent with modification of PE whereas the level of CFA protein stayed relatively flat at late time points. (C) As represented here by the 6 h sample, lipid A from all samples collected between 3 and 48 h contained one major peak at ~1797 m/z corresponding to wild type, hexa-acylated lipid A. As illustrated on the right by the two week sample, the one and two week (168 and 336 h) time points showed the addition of the C_16_ chain to lipid A. (D) Phosphatidylethanolamine (PE) is shown and similar results were also obtained for phosphatidylglycerol (PG). The phospholipid profiles of the samples remained relatively consistent with wild-type *E*. *coli* phospholipid profiles until hour 8, when a gradual increase in a peak ~702.5 m/z began. This peak became the predominant species by two weeks. Its mass corresponds to the cyclopropanation of one unsaturated double bond within a PE molecule containing acyl chains totaling 33 carbons distributed between the two acyl chains.

Using negative-ion MALDI-TOF and ESI mass spectrometry (MS), we analyzed lipid A and phospholipid profiles, respectively, of cells at each time point. Beginning before one week, we observed an appearance of an MS peak associated with the acylation of lipid A with a C_16_ chain (Fig 5A and 5C). In the phospholipid analysis, a notable increase began around 6 h in the cyclopropanation of one unsaturated double bond within molecules of the major phospholipids, phosphatidylethanolamine (PE) and phosphatidylglycerol (PG). This change was identified by the gradual relative increase of peaks at ~702.5 m/z and ~733.5 m/z, respectively. (Representative data for PE is shown in Fig 5D.) Both the modifications to lipid A and phospholipids continued to increase up to the two-week time point. In fact, the 702.5 m/z peak corresponding to cyclopropanation of phospholipid was barely detectable before six hours but became the predominant peak by the end of the time course.

The enzymes relevant to the above lipid A and phospholipid modifications are lipid A palmitoyl transferase (PagP) and cycloproponated fatty acid synthase (CFA), respectively [31,32]. PagP is known to be constitutively transcribed at low levels and remain latent in the outer membrane until enzyme activation [33]. It is also up-regulated by the transcriptional regulator, PhoP, under various stressful conditions encountered by a cell [34]. However, during our time course, transcript levels of PagP and PhoP did not change significantly. Furthermore, neither PagP nor PhoP was observed at the protein level. In the case of PagP, this could be due to the difficulty in detecting outer membrane beta-barrel proteins by our mass-spec proteomics method. With respect to phospholipid modification, CFA synthase protein levels increased between 3–6 h before decreasing again. This observation agreed with prior data showing that CFA synthase was important during the transition to stationary phase [32]. CFA synthase RNA levels increased again around one week, which was consistent with the activity observed in phospholipid modification, although it is not clear why we did not observe a corresponding increase in protein levels at this point (Fig 5B).

## Discussion

We have collected a comprehensive *E*. *coli* time course and have developed computational techniques to analyze such data. Our computational techniques are general and can be applied to other time-course data collected in future studies. In particular, fitting piecewise continuous curves to expression profiles allowed us to reliably sort individual profiles into four basic groups, up-regulated, down-regulated, transiently up-regulated, or transiently down-regulated. Additionally, we have developed an unbiased approach to compare mRNA and protein profiles and to identify those proteins whose abundances followed their mRNA levels and those that were buffered against rapid mRNA changes.

**Fig 6:**
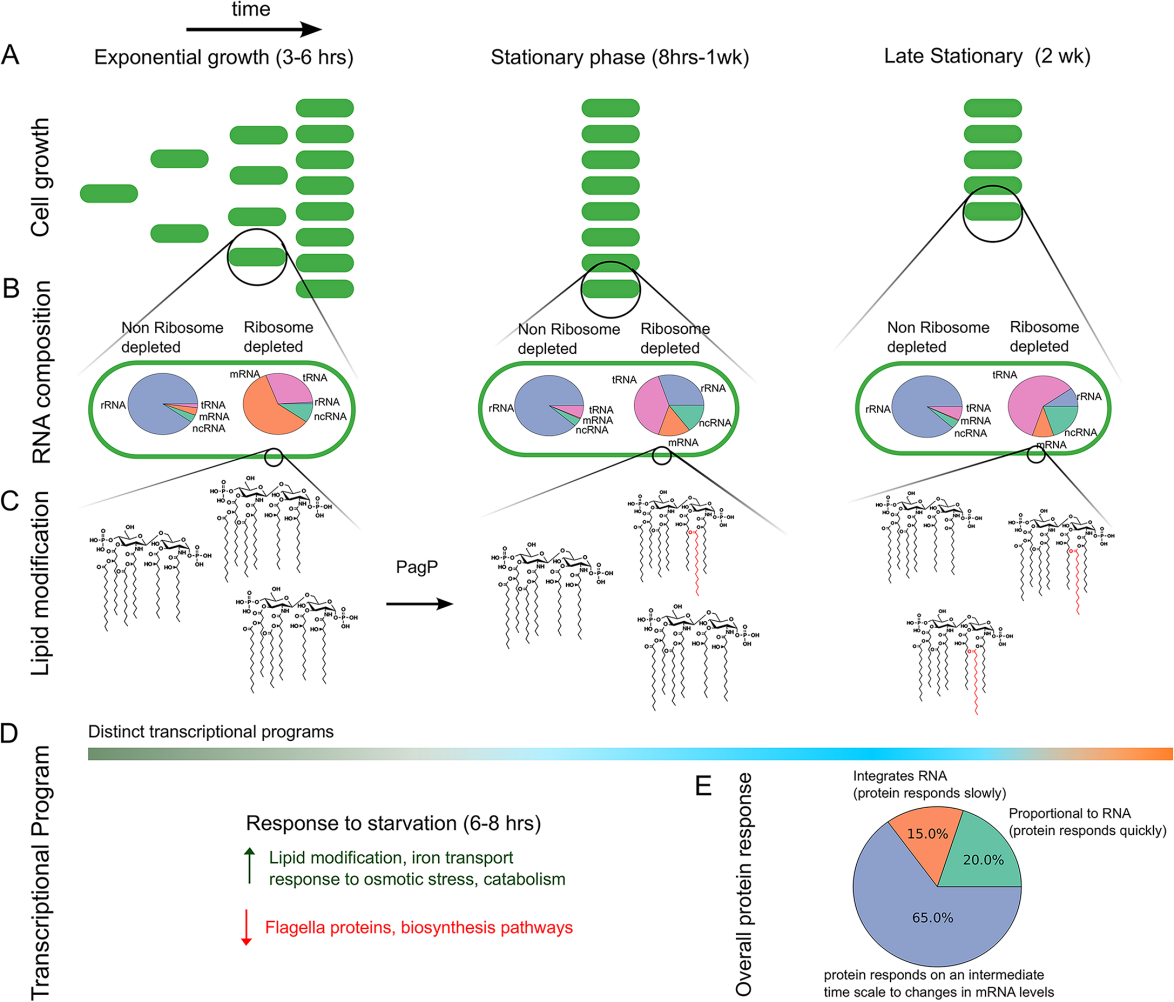
Summary of key results. (A) Cells are starved around 6–8 h after the initiation of growth, and the cell viability (and OD_600_) remained constant up until at least one week. At two weeks, however, there was a decrease of 38% in viability. (B) The relative fraction of rRNA (compared to all RNA) stayed fairly constant through the entire time course, as did the fraction of tRNA (in non-rRNA depleted samples). On the other hand, relative levels of mRNA decreased upon entry to stationary phase perhaps as a strategy for reducing overall protein synthesis. As a reference we also analyzed RNA fractions for the rRNA-depleted samples, to demonstrate that this reduction of mRNA levels was not simply due to the low relative counts of mRNA compared to rRNA in the nondepleted samples. Note that the rRNA-depleted samples still contained residual amounts of rRNA, as shown. (C) Phospholipids and lipid A were modified in a manner consistent with the activation of stress responses. Modifications began early in the stationary phase and slowly increased during the time course for up to two weeks. (D) Transcriptional changes (measured by mRNAs) separated into at least two temporal domains, before and after stationary phase. A possible third region corresponded to late transcriptional changes observed at two weeks. All changes in regulation had begun by 10 h. At this point stress response genes were up-regulated and energy intensive processes were down-regulated. (E) Approximately 20% of the measured protein levels were proportional to their transcript levels over time while 15% of the protein levels integrated their transcript’s response over the entire duration of the experiment. This observation highlights that differences in post-transcriptional regulation, such as protein degradation, cause differences in regulation between mRNAs and their expressed proteins.

Our results provide a coherent picture of *E*. *coli* stationary phase, as summarized in Fig 6. *E*. *coli* could survive for over a week when starved for glucose in a well-buffered minimal medium, with little change in cell viability (Fig 6A). The fraction of mRNA relative to all RNA was down regulated after cells entered stationary phase (Fig 6B). As cells ceased to divide, the demand for new protein synthesis declined. Reducing the overall pool of mRNA could contribute to limiting new protein synthesis. Upon entry to stationary phase, lipid A and phospholipids were modified by PagP and CFA synthetase, respectively (Fig 6C). Modification of lipids continued gradually until eventually the lipid species that were rare during growth dominated at two weeks.

All genes started to change in expression by 10 h, and mRNA expression clustered temporally into two regimes, before and after 10 h (cells entered a starved state at around 8 h) with some late changes in expression beginning around two weeks (Fig 6D). We found that 20% of observed proteins were regulated in proportion to their transcripts (Fig 6E), allowing for rapid down-regulation of the processes they were involved in. On the other hand, 15% of the observed proteins were integrally related to their transcripts (Fig 6E) and likely served to buffer against environmental changes. In addition to measuring and characterizing RNA and protein changes upon entry to stationary phase, we also demonstrated how a piecewise curve-fitting strategy allowed us to classify expression profiles into different categories. The enriched terms in the resulting classification were reasonably aligned with what was known about, or at least consistent with, cells coping with starvation (Fig 6D). Importantly, this classification was accomplished in an unbiased manner, without any ad hoc assumptions about the number of clusters that should exist in the data.

We found that, as cells entered a starved state, the total pool of mRNA was depleted compared to all other RNAs and many individual transcripts were down-regulated, possibly as part of a broader strategy to reduce the production of new protein. Reducing overall protein production could also be achieved by limiting the available ribosomes or by limiting the pool of available tRNA. The stringent response, activated in starving cells through the ppGpp alarmone, down-regulates new rRNA synthesis [35]. However, in our data, the relative fraction of rRNA within a cell changed little over time, and the tRNA fraction actually increased with time. Thus, new protein synthesis in starving cells may be limited more by the reduced mRNA pool than by reduced translational efficiency due to decreases in rRNA or tRNA abundance. Even if the total rRNA decreased over the time course, the total mRNA would have decreased more by a proportional amount. Said another way, the down-regulation of new rRNA synthesis by the stringent response may be most important for shutting down the production of ribosomes needed by new cells in an actively dividing culture, rather than for reducing the level of ribosomes in already existing cells.

It has been suggested that the degradation rate of many proteins in *E*. *coli* is much slower than the doubling time during growth [36,37]. As a consequence, when cells cease to divide, such as in the case of glucose starvation, not all proteins can respond immediately to possible changes in transcript levels. In effect, the amounts of some proteins may be buffered against relatively fast changes in nutrient availability. At the same time certain proteins may need to be rapidly regulated to ensure survival upon starvation. We found that a subset of the proteome, ~20% of proteins, fell into the rapidly regulated category that may be degraded quickly—they maintained an abundance that was proportional to their transcripts. Another subset, ~15% of proteins, tended to be much more stable—they were proportional to the integrated abundance of their transcripts over the time scale of our experiment. For example, the abundance of several flagellar proteins was proportional to their transcript levels, whereas proteins involved in metabolism and energy production integrated their transcript levels over time. Turning off proteins involved in cell division and the flagellar machinery, both energy-intensive processes, needs to happen relatively quickly. By contrast, the proteins that were relatively stable were enriched for energy production terms. Thus, these proteins presumably persist so that if nutrients were to become available again the cell will be capable of using them to re-initiate growth. For proteins to track dynamically with their transcripts they must have a short half-life. For this reason, we can compare those terms enriched for proteins that dynamically correlate with their transcripts to the COG terms reported by Maier et al. [23] that have shorter than average half lives in *M*. *pneumoniae*. We found that those COG terms with shorter than average half-lives were generally consistent with terms that were enriched in highly dynamically correlated proteins and mRNAs. In particular, Maier et al. found that terms involved with energy production (COG term C), metabolism (COG terms H, I, G), protein turnover (COG term O), and signaling (COG term T) had protein turnover rates significantly faster than the overall average.

Among the terms that were significantly regulated in stationary phase, we saw that motility was down-regulated, likely because it places a high energy burden on cells [1]. Additionally, it has been shown that flagella in *E*. *coli* are down-regulated by the stringent response [38]. Other observed differential regulation is related to energy conservation (shutting down expensive or unneeded pathways), catabolism (breaking down non-essential components for food), stopping translation of new protein (as there is no longer demand for protein from new cells), or a general stress response (increasing nutrient influx or bolstering membrane integrity).

We also found many uncharacterized genes (both among the protein and the RNA profiles) that were significantly up- or down-regulated upon entry to stationary phase. A subset of these proteins have computationally predicted functions [39] that were consistent with our findings for annotated genes. For instance, several uncharacterized proteins that were up-regulated are predicted to be involved in stress response and cell-wall biogenesis. Other predictions seem to be inconsistent with our observations for annotated genes or indicate that these genes regulate rather than take part in these processes. For example, some uncharacterized proteins that were up-regulated are predicted to be involved in translation, even though translation was heavily enriched in down-regulated genes. These uncharacterized genes might down-regulate the activity of ribosomes, for example. Lists of proteins and transcripts that were significantly regulated in our time course are provided in the Supplemental materials (S3 and S4 Tables).

Even though mRNA abundances within an operon were highly correlated (as expected), in many cases their protein profiles were only weakly correlated. This finding could be due to different translation efficiencies between proteins [40] as well as differing degradation rates. In support of the former, we saw a tendency for proteins separated by a larger distance within a transcript to be less correlated than those located closer to one another. However, it was likely that different protein degradation rates also played a role in the low correlation between proteins within an operon. Indeed, many proteins coded by proximal regions of a transcript showed poor correlation in their profiles (Fig 3C). Other explanations for this tendency of proteins nearby on the genome to be more highly correlated could be due to distance from the transcript start site or transcript length. Yet, our data did not show evidence for either of these scenarios. Distance from the transcript start site was not correlated with protein expression (ρ = –0.02, *P* = 0.65) and transcript length was only very weakly correlated with protein expression (ρ = 0.12, *P* = 0.003). However, we cannot necessarily rule out other explanations for the observed intra-operon protein correlation vs. distance between genes.

In addition to the expected disparities between RNA and protein levels, we also observed surprising changes in enzyme activity that did not correspond to the respective RNA-seq and proteomics analysis. For example, we saw striking levels of lipid modification late during the time course. These modifications were easily explained by their association with adaptation to stressful environments such as depleted nutrients and cations as well as increased acid resistance during starvation [32,34]. However, the stark differences in RNA, protein, and activity trends of the enzymes responsible for the lipid modifications, PagP and CFA synthase, highlight the fact that activation does not necessarily follow abundance measurements. In support of this idea, it has been shown that cylopropination by CFA synthase depends upon the concentration of bicarbonate, which could lead to a decoupling between protein levels and activation [41].

Metabolic fluxes were quite constant throughout the growth phase of the experiment, and these labeling patterns remained in place once growth ceased. At the two-week time point, however, the labeling patterns in histidine changed substantially, which during steady-state growth on glucose would have been interpreted as a change in the flux ratio corresponding to P5P from the G6P lower branch declining. This change so late in the experiment was unexpected, since we did not anticipate substantial turnover in cellular composition that late in stationary phase. The observation suggests that either internal amino acid recycling or some *de novo* amino-acid synthesis, possibly related to the moderate decline in the number of viable cells, occurs past the one week time point.

A goal of systems biology has been to understand how phenotype originates from genotype. The phenotype of a cell is determined by complex regulation of processes including cell signaling, gene regulation, metabolism, and lipid biochemistry. Understanding the connection between phenotype and genotype is crucial to understanding disease and for synthetic engineering of biology. Even though computational models of individual component subsystems, such as flux models of metabolism [42–44], have enjoyed a long history of success, they remain limited in their application. Much effort is currently being spent on understanding how to best integrate data from multiple subsystems. For example, there are many proposed approaches to combining gene expression with metabolic flux networks [20,45–52] while other studies have focused on integrative, whole-cell models [53,54]. Given the growing interest in integrative modeling approaches, there is a pressing need for studies that collect high quality genome-wide data across multiple cellular subsystems from the same biological samples. Our data set is a rich resource for comparing and contrasting the response of multiple cellular subsystems. Additionally, in the future we plan to use the techniques developed in this paper to measure the response of *E*. *coli* to several other environmental conditions, which will allow for more detailed models of regulation.

Despite the completeness and quality of our data set, however, there were a few key limitations concerning our approach. Our analysis via RNA-seq and shotgun MS allowed for high confidence when comparing the relative levels of a particular transcript or protein over time. However, due to potential differences in detection efficiency between individual RNAs or peptides, care should be taken when comparing absolute abundances. In our analysis we used the APEX method to account for differences in protein detection efficiency. We normalized RNA by the length of a transcript as an estimate of RNA detection efficiency, for a particular experiment. This approach resulted in a correlation coefficient of ~0.71 between proteins and their transcripts, a finding on the high end for such correlation measurements. Previous reports on the correlation between mRNA and protein levels in *E*. *coli* and *M*. *pneumoniae* have yielded correlation coefficients of ~0.5 [18–23]. Thus, even straightforward means of correcting our experimental bias led to reasonable comparisons of levels between individual RNAs or proteins. Additional detection biases we did not account for, such as GC content bias in RNA-seq [55], were likely responsible for some of the remaining unexplained variation [56].

The stepwise linear function we used for modeling works for a majority of our expression profiles. However, in some cases it over-fits the data and in other cases the function is unable to capture the underlying behavior. An example of a profile that may be under-constrained is a gene that is up- or down-regulated without further changes to expression. In this case the free time parameters *t*_2_ through *t*_4_, along with amplitude parameter *A*_2_, may be under-constrained. Even in this case, however, the parameters *t*_1_, *t*_2_+*t*_3_+*t*_4_, *A*_1_, and *A*_3_ are still well constrained, providing enough information to reliably sort the profiles based upon behavior. Thus, since we used our model for classification and not for prediction purposes, any potential parameter over-fitting did not substantially affect our final results. More complicated temporal profiles, such as multiple peaks separated in time, could not be captured by our function. The presence of these more complicated behaviors was rare enough as to not warrant special consideration.

In summary, our study provides both the most complete measurement, to our knowledge, of multiple cellular components in a changing environment, and novel computational approaches to analyze such data. Thus, this work represents an important step toward understanding how regulation of a cell’s physiology is coordinated, on a global, systems level, by interactions between multiple cellular subsystems.

## Methods

### Cell growth

*E*. *coli* B REL606 was inoculated from a freezer stock into 10 ml of Davis Minimal medium supplemented with 2 μg/l thiamine (DM) [57] and limiting glucose at 500 mg/l (DM500) in a 50 ml Erlenmeyer flask. This culture was incubated at 37°C with 120 r.p.m. orbital shaking over a diameter of 1". After overnight growth, 500 μl of the culture was diluted into 50 ml of prewarmed DM500 in a 250 ml flask and grown for an additional 24 h under the same conditions. On the day of the experiment, 500 μl of this preconditioned culture was added to ten 250 ml flasks, each containing 50 ml DM500, to initiate the experiment. At each time point, aliquots of these cultures were removed as necessary to harvest a constant number of cells given the changes in cell density over the growth curve. Each sample was pelleted by centrifugation, washed with sterile saline (0.85% (w/v) NaCl), and then spun down again. After removing the supernatant, the resulting cell pellet was flash frozen using liquid nitrogen and stored at –80°C. Each of the three biological replicates was performed on a separate day. Samples for each type of cell composition measurement were taken from the same batch of flasks, except for those used for flux analysis, for which an additional batch was grown in 20% [U-^13^C] glucose.

For graphs of OD_600_ and colony-forming units (CFU), cultures were grown separately from the main batches used for harvesting cells but under identical conditions. The OD_600_ (absorbance at 600 nm) of a sample removed from the culture at each time point was measured relative to a sterile DM500 glucose blank. These samples were also diluted in sterile saline and plated on DM agar supplemented with 0.2 g/l glucose. After incubation at 37°C for 24 h colonies on these plates were counted to determine CFUs.

### RNA-seq

Total RNA was isolated from cell pellets using the RNAsnap method [2]. After extraction, RNA was ethanol precipitated and resuspended in 100 μl H_2_O. Each sample was then DNase treated and purified using the on-column method for the Zymo Clean & Concentrator-25 (Zymo Research). RNA concentrations were determined throughout the purification using a Qubit 2.0 fluorometer (Life Technologies). DNase-treated total RNA (≤5 μg) was then processed with the Gram-negative bacteria RiboZero rRNA removal kit (Epicentre). After rRNA depletion, each sample was ethanol precipitated and resuspended in H_2_O again. A fraction of the RNA was then fragmented to ~250 bp using NEBNext Magnesium RNA Fragmentation Module (New England Biolabs). After fragmentation, RNA was ethanol precipitated, resuspended in 20 μl ultra-pure water, and phosphorylated using T4 PNK (New England Biolabs). After another ethanol precipitation cleanup step, sequencing library preparation was performed using the NEBNext Small RNA Library Pre Set for Illumina, Multiplex Compatible (New England Biolabs). Samples were ethanol precipitated again after library preparation and separated on a 4% agarose gel. All DNA fragments greater than 100 bp were excised from the gel and isolated using the Zymoclean Gel DNA Recovery kit (Zymo Research). Libraries were sequenced using an Illumina HiSeq 2500 at the Genomic Sequencing and Analysis Facility (GSAF) at the University of Texas at Austin to generate 2×101-base paired-end reads.

For RNA-seq analysis, we implemented a custom analysis pipeline using the REL606 *Escherichia coli* B genome (GenBank:NC_012967.1) as the reference sequence [58]. We updated annotations of sRNAs in this genome sequence using the Rfam 11.0 database [59]. Prior to mapping, we trimmed adapter sequences from Illumina reads using Flexbar 2.31 [60]. Mapping was carried out in single-end mode using Bowtie2 2.1.0 with the –k 1 option to achieve one unique mapping location per read [61]. The raw number of reads mapping to each gene was counted using HTSeq 0.6.0 [62]. The full computational pipeline is available at https://github.com/wilkelab/AG3C_starvation_tc_RNAseq.

### Proteomics

*E*. *coli* cell pellets were resuspended in 50 mM Tris-HCl pH 8.0, 10 mM DTT. 2,2,2-trifluoroethanol (Sigma) was added to 50% (v/v) final concentration and samples were incubated at 56°C for 45 min. Following incubation, iodoacetamide was added to a concentration of 25 mM and samples were incubated at room temperature in the dark for 30 min. Samples were diluted 10-fold with 2 mM CaCl_2_, 50 mM Tris-HCl, pH 8.0. Samples were digested with trypsin (Pierce) at 37°C for 5 h. Digestion was quenched by adding formic acid to 1% (v/v). Tryptic peptides were filtered through Amicon Ultra 30 kD spin filtration columns and bound, washed, and eluted from HyperSep C18 SpinTips (Thermo Scientific). Eluted peptides were dried by speed-vac and resuspended in Buffer C (5% acetonitrile, 0.1% formic acid) for analysis by LC-MS/MS.

For LC-MS/MS analysis, peptides were subjected to separation by C18 reverse phase chromatography on a Dionex Ultimate 3000 RSLCnano UHPLC system (Thermo Scientific). Peptides were loaded onto an Acclaim C18 PepMap RSLC column (Dionex; Thermo Scientific) and eluted using a 5–40% acetonitrile gradient over 250 min at 300 nl/min flow rate. Eluted peptides were directly injected into an Orbitrap Elite mass spectrometer (Thermo Scientific) by nano-electrospray and subject to data-dependent tandem mass spectrometry, with full precursor ion scans (MS1) collected at 60,0000 resolution. Monoisotopic precursor selection and charge-state screening were enabled, with ions of charge >+1 selected for collision-induced dissociation (CID). Up to 20 fragmentation scans (MS2) were collected per MS1. Dynamic exclusion was active with 45 s exclusion for ions selected twice within a 30 s window.

Spectra were searched against an *E*. *coli* strain REL606 protein sequence database and common contaminant proteins (MaxQuant using SEQUEST (Proteome Discoverer 1.4; Thermo Scientific)). Fully-tryptic peptides were considered, with up to two missed cleavages. Tolerances of 10 ppm (MS1) and 0.5 Da (MS2), carbamidomethylation of cysteine as static modification, and oxidized methionine as dynamic modification were used. High-confidence peptide-spectral matches (PSMs) were filtered at <1% false discovery rate determined by Percolator (Proteome Discoverer 1.4; Thermo Scientific).

### Flux analysis

Flux ratios were obtained from the samples grown with ^13^C labeled glucose, using methods previously described [28,29]. Cell pellets were resuspended in 200 ml of 6 N HCl, hydrolyzed at 105°C overnight, and dried at 95°C for up to 24 h. To the hydrolyzed cell material we added 40 ml of dimethylformamide (DMF) and gently mixed until a “light straw” color was obtained. The DMF resuspension was transferred to a GC-MS vial with plastic insert and 40 ml of *N*-*tert*-butyldimethylsilyl-*N*-methyltrifluoroacetamide with 1% *tert*-butyldimethyl-chlorosilane (v/v); vials were capped and baked at 85°C for 2 h, and samples were analyzed within 2 days of derivitization.

Analysis of derivitized samples was performed on a Shimadzu QP2010 Plus GC-MS (Columbia, MD) with autosampler. The GC-MS protocol included: 1 mL of sample injected with 1:10 split mode at 230°C; an oven gradient of 160°C for 1 min, ramp to 310°C at 20°C/min, and hold at 310°C for 0.5 min; and flow rate was 1 mL/min in helium. A total of five runs were performed for each sample: a blank injection of DMF to waste, a blank injection of DMF to the column, and three technical replicates of each vial.

Flux inference was performed using the FiatFlux software as described [29,30]. Each time point was analyzed separately, and the reported fluxes represent the integral of growth up until that point. For this reason, we do not display fluxes beyond 8 h, when growth ceased and there should not be any more net amino acid synthesis. We did, however, monitor the labeling patterns in all amino acids for the later time points. Although most patterns were unchanged, we did note that histidine labeling changed substantially at the final time point of two weeks.

### Lipid analysis

Lipid A and phospholipids were isolated from bacterial pellets containing 3–9×10^9^ cells. Pellets were resuspended in 5ml 1:2:08 chloroform:methanol:water for 20 min and spun at 10,000×g for 10 minutes. Pellets containing lipid A were further purified by the Bligh/Dyer method as previously described [63]. Phospholipids in the supernatant were further purified by extractions as previously described [64]. Mass analysis of purified lipid A fractions was performed using a MALDI-TOF/TOF (ABI 4700 Proteomics Analyzer) mass spectrometer in the negative ion linear mode as previously described [63]. Phospholipid analysis was performed by liquid chromatography/ESI-mass spectrometry as previously described [64]. One of the three replicates used for lipid analysis was an additional independent biological replicate, prepared identically to all other replicates but not used for RNA-seq or proteomics analysis.

### Expression profile data analysis

We analyzed raw counts from the proteomics and RNA-seq experiments as follows. Initially, proteins with low counts (<10) over the entire duration of the time course were filtered out. Each time point was then normalized to the read depth (e.g. the sum of all counts for that particular time point). Only proteins with a fold change of ≥1.5 were considered for further analysis. Protein profiles were then normalized to the maximum value for a given protein time course. To estimate the absolute protein abundance we made use of the APEX normalization method [65].

To analyze relative RNA levels, raw RNA read counts per gene (ignoring rRNAs) were normalized within each sample using DESeq [16]. To identify RNAs that had changed significantly, we carried out a differential expression analysis between the 3 h time point and each subsequent time point, using DESeq, and we kept RNAs with a significant difference (*p* < 0.05) at least one time point for further analysis. To compare absolute RNA abundances within a single time point, raw RNA counts were normalized by gene length. Finally, normalized RNA and protein profiles, both relative and absolute, were averaged across all three biological replicates.

Clustering of protein profiles was performed using the python library scipy [66]. We used the *k*-means clustering algorithm with the number of protein clusters set to 25 and RNA clusters to 15. To compare relative protein profiles with the integral of their relative transcript levels we integrated each of the transcript profiles, from the initial time to each additional time point, using the trapezoidal method implemented by the python library numpy [67].

We used a piecewise continuous curve to fit both RNA and protein profiles. This curve was defined by seven free parameters, four free time parameters, and three free amplitude parameters. To fit the profiles we used a custom implementation of a differential evolution (DE) algorithm [68]. Briefly, the DE algorithm initially generates an ensemble of random parameter guesses within a predefined range; subsequently, vectors of individual parameter sets (sometimes called agents) are mixed together at a predefined crossover rate, only those crossover events that yield a smaller error (defined by a predefined cost function) are kept, and the process is iterated until a convergence criterion is met. In our fits we used an ensemble of 15 agents with a crossover frequency of 0.75 and a mixing strength of 0.6. The crossover frequency determines the probability that an agent will be changed at any given iteration and the mixing strength determines how large a change an agent undergoes if it was chosen to be altered. The crossover frequency and mixing strength were picked based upon an empirical study of the dependence of convergence efficiency on these parameters [69] for some standard optimization problems. The cost function is given by

$$F_{i}=\sum_{j}\frac{{\left(d_{i}(t_{j})-s_{i}(t_{j})\right)}^{2}}{\sigma_{i}{}^{2}(t_{j})}$$

where *d*_*i*_(*t*_*j*_), *σ*_*i*_(*t*_*j*_), and *s*_*i*_(*t*_*j*_) are the average of all experimental repeats of protein (or mRNA) *i* at time *t*_*j*_, the standard deviation of the experiments of the protein (or mRNA) *i* at time *t*_*j*_, and the average of the ensemble simulations *i* at time *t*_*j*_, respectively. Scaling by the standard deviation places a relatively lower weight on data points with relatively larger errors for a given protein or mRNA.

Some of the profiles may be slightly over-fit by our curve (e.g. profiles that are up-regulated or down-regulated once during the time course without further modulation of expression). Thus care needs to be exercised in the interpretation of some of the parameters. However, we found *t*_1_ to reliably represent the time to first inflection, the sum of *t*_2_, *t*_3_, and *t*_4_ was a decent proxy to how long it took an RNA/protein to reach a steady state after entering a starved state, and we could reliably sort the behavior into four categories based upon the amplitude parameters. The four categories we used were that of up-regulated, down-regulated, transiently up-regulated, and transiently down-regulated. Genes that were up (or down) regulated were those genes that increased (or decreased) at some point during the time course and did not decrease (or increase) at some later time. Genes that were transiently up (or down) regulated were those genes that increased (or decreased) at some point during the time course but decreased (or increased) at some later time. The sorting into categories was aided by our estimate of the distribution of parameters that allowed for a good fit within the population of fits. A fit was considered good if it was on average (across the time course) one standard deviation or less away from the experimental average.

We used the DAVID database (david.abcc.ncifcrf.gov) to perform Gene Ontology term enrichment on each subset of sorted genes: up-regulated, down-regulated, transiently up-regulated, and transiently down-regulated. Specifically we made use of DAVID's API, instead of the web interface, to generate the GO-enrichment through a python script. GO terms were clustered based upon genes in a given term to reduce redundancy in the returned results.

As a complementary approach we also enriched for KEGG pathway terms in the entire set of significantly changing proteins (without presorting) using the DAVID database API. The protein levels within each returned KEGG pathway were then averaged to see if there was any consistent response across the entire pathway. Those KEGG terms that gave inconsistent responses across proteins in that pathway returned a relatively flat average and were filtered out.

All of the scripts used to perform the above analysis can be downloaded at https://github.com/marcottelab/AG3C_starvation_tc.

### Data availability

Raw data are available from the Dryad Digital Repository: http://dx.doi.org/10.5061/dryad.hj6mr. Raw Illumina read data and processed files of read counts per gene and normalized expression levels per gene have been deposited in the NCBI GEO database (accession GSE67402) [70]. The mass spectrometry proteomics data have been deposited to the ProteomeXchange Consortium via the PRIDE partner repository (accession PXD002140) [71].

## Supporting Information

S1 Table Functional clustering of proteins that are proportional to their transcripts. (CSV)

S2 Table Functional clustering of proteins that are proportional to the integral, over time, of their transcripts. (CSV)

S3 Table List of proteins that are significantly changing throughout the time course. For each gene, we list the common name, the entrez ID, the full-length gene name, the predicted function for uncharacterized genes, and the behavioral group each gene was sorted into (up-regulated, down-regulated, temporally up-regulated, temporally down-regulated, ambiguous/unsorted).
(CSV)

S4 Table List of RNAs that are significantly changing throughout the time course. For each gene, we list the common name, the entrez ID, the full-length gene name, the predicted function for uncharacterized genes, and the behavioral group each gene was sorted into (up-regulated, down-regulated, temporally up-regulated, temporally down-regulated, ambiguous/unsorted).
(CSV)

S1 Fig RNA-seq and MS experiments were highly reproducible. Scatter plot between biological replicates 1 and 2 (protein and RNA, left column) and 1 and 3 (protein and RNA, right column) along with their associated Spearman correlation coefficients. P-values for all correlations are <10^−100^.
(TIFF)

S2 Fig Overlap in protein IDs between first three time points. Overlap between protein IDs comparing the first three time points, 3–5 hrs, where cells and protein concentrations are roughly at steady state. The high overlap between time points indicates very reproducible protein IDs.
(TIFF)

S3 Fig The mRNA fraction, compared to all other RNA, was strongly down-regulated after entry to stationary phase. For each time point the fraction of total RNA reads in the RNA-seq results that mapped to tRNA (orange), rRNA (green), mRNA (red), or other noncoding RNA (purple) are shown. (A) RNA fractions for each total RNA sample that was processed without the rRNA depletion step. (B) RNA fractions for rRNA-depleted samples. Each bar represents an individual biological repeat. In some samples the rRNA depletion was not as successful as in others (e.g., biological replicate 1 at the 8 h time point). Any residual rRNA counts were disregarded before analyzing relative RNA expression levels.
(TIFF)

S4 Fig Correlations between the absolute abundance between protein and mRNA was strongest at 3 hrs. Both the RNA and protein levels were scaled to their respective averages across all RNAs or proteins for each time point and then log transformed. All P values were <10^−43^.
(TIFF)

S5 Fig Fitting of piecewise continuous curve was effective when sorting response curves. We grouped RNA and protein time courses based on general qualitative behaviors. After entry to stationary phase, RNA and/or protein can be shut off, turned on, transiently activated, or transiently repressed. (A) To sort the profiles, a piecewise continuous curve was fit to the data. The parameter *t*_0_ represents the time at which we start to collect data at 3 h into growth. The curve was fit using a differential evolution fitting algorithm that was gradient free and population based, allowing for a range of possible parameter sets that can explain our data given the experimental error. (B-E) Four random examples of measured RNA time courses averaged across 3 biological replicates (green circles) with their standard deviations (green bars) along with the corresponding fits (blue). The blue bars represent the standard deviation of the range of fits that agree with our data. Both experimental time courses and fits were normalized by the average of the time course. (F) Most of the RNAs began to change between 6–8 h, when the cells began to be starved. This is demonstrated by the histogram of *t*_1_, the time to the first inflection point.
(TIFF)

S6 Fig Distributions of time scales found by fitting the piecewise continuous curve, described in the main text, to the mRNA and protein profiles. (A-C) mRNA distributions of *t*_1_, time to first inflection (A), *t*_2_ + *t*_3_ + *t*_4_, the time between the first inflection and time the profile levels off (B), and *t*_1_ + *t*_2_ + *t*_3_ + *t*_4_, the total time until a given profile levels off (C). (D-F) As (A-C), but for protein profiles.
(TIFF)

S7 Fig Flux ratio profiles in long-term glucose growth. Flux ratios were computed via the FiatFlux software from GC-MS derived ^13^C constraints. As FiatFlux considers each time point as an integral from the start of the experiment, this analysis allowed us to determine whether later time points during growth changed the overall central metabolic flux splits that were estimated from earlier time points. Flux ratios for (A) SER from GLY, (B) OYR from MAL upper branch, (C) PEP through TK upper branch, (D) PEP through PPP upper branch, (E) PEP from OAA, (F) OAA from PEP, (G) P5P from G6P lower branch, (H) E4P through TK, and (I) GLY through serine.
(TIFF)

S1 File Raw RNA and protein counts, flux ratio data, lipid spectra, and phospholipid spectra. (ZIP)

S2 File Normalized and averaged, across biological replicates, RNA and protein counts. (ZIP)

S3 File Functional clustering of GO enrichments for protein time course profiles sorted into behavioral groups. (ZIP)

S4 File Functional clustering of GO enrichments for RNA time course profiles sorted into behavioral groups. (ZIP)
