# Supplementary figures and images for: Controlled Measurement and Comparative Analysis of Cellular Components in E. coli Reveals Broad Regulatory Changes in Response to Glucose Starvation
Source: PLoS Comput Biol. 2015 Aug 14;11(8):e1004400. doi: 10.1371/journal.pcbi.1004400 (PMC4537216; doi:10.1371/journal.pcbi.1004400)

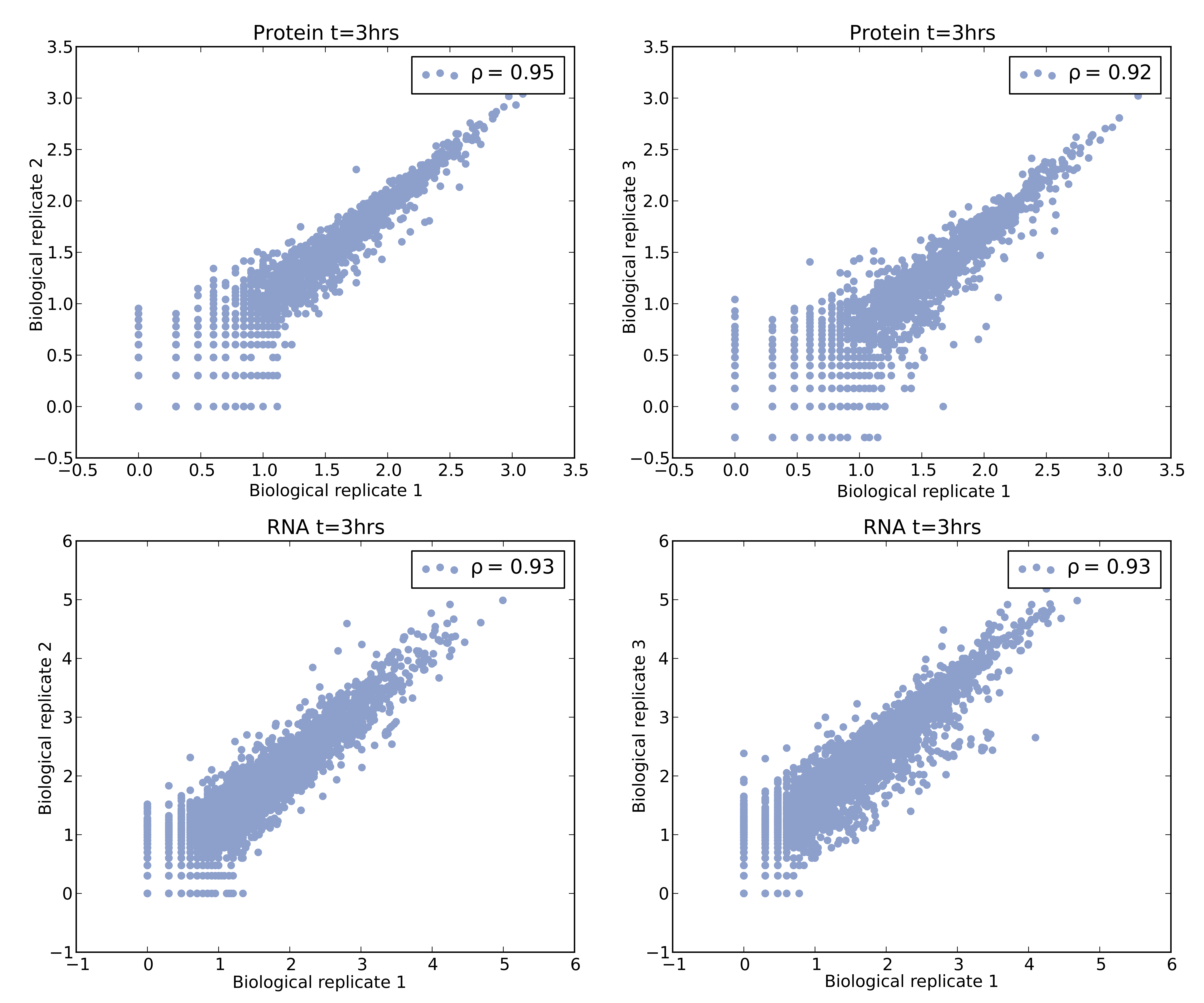

Supplement: S1 Fig — Scatter plot between biological replicates 1 and 2 (protein and RNA, left column) and 1 and 3 (protein and RNA, right column) along with their associated Spearman correlation coefficients. P-values for all correlations are <10−100. (TIFF) [file pcbi.1004400.s005.tiff]

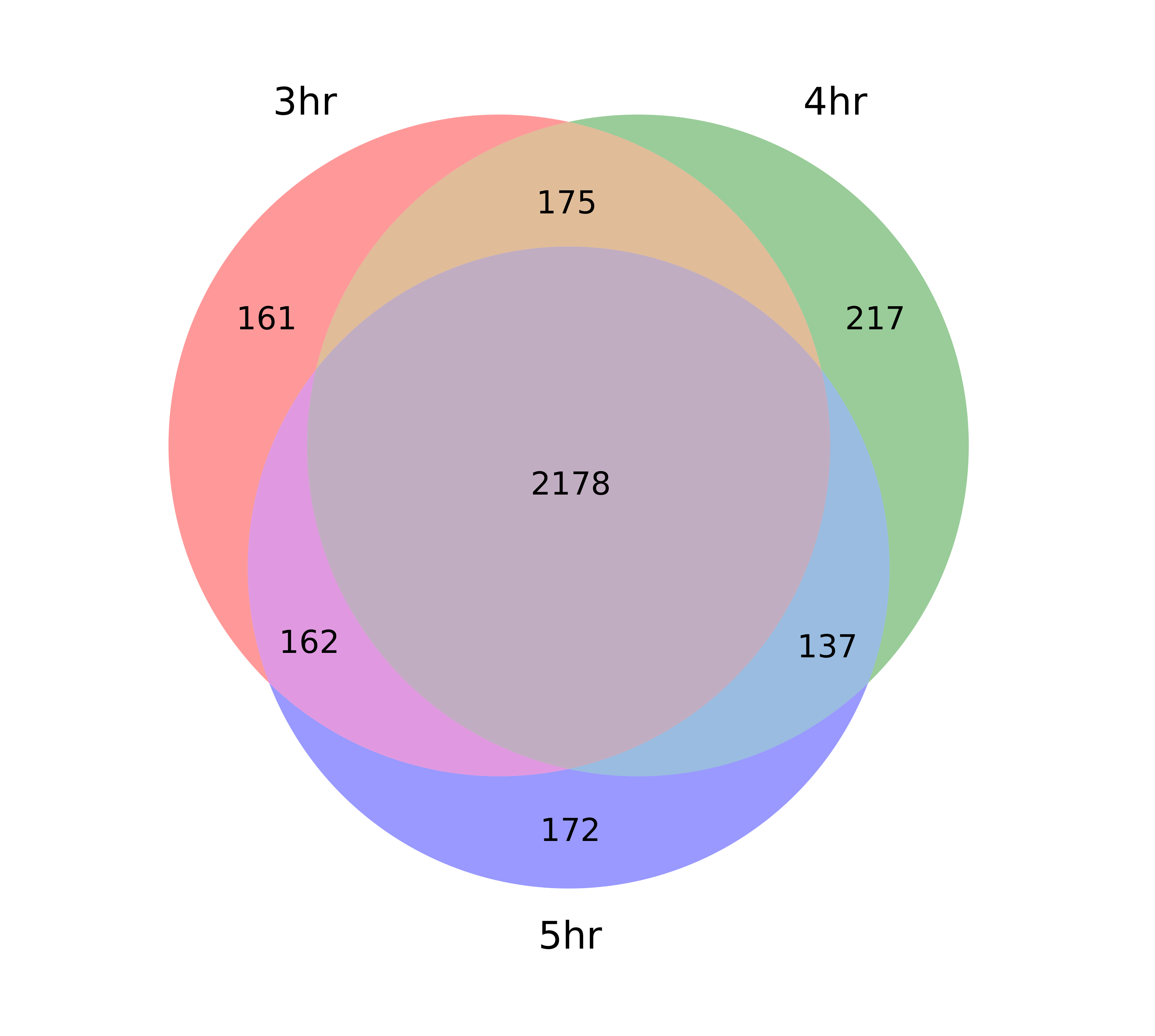

Supplement: S2 Fig — Overlap between protein IDs comparing the first three time points, 3–5 hrs, where cells and protein concentrations are roughly at steady state. The high overlap between time points indicates very reproducible protein IDs. (TIFF) [file pcbi.1004400.s006.tiff]

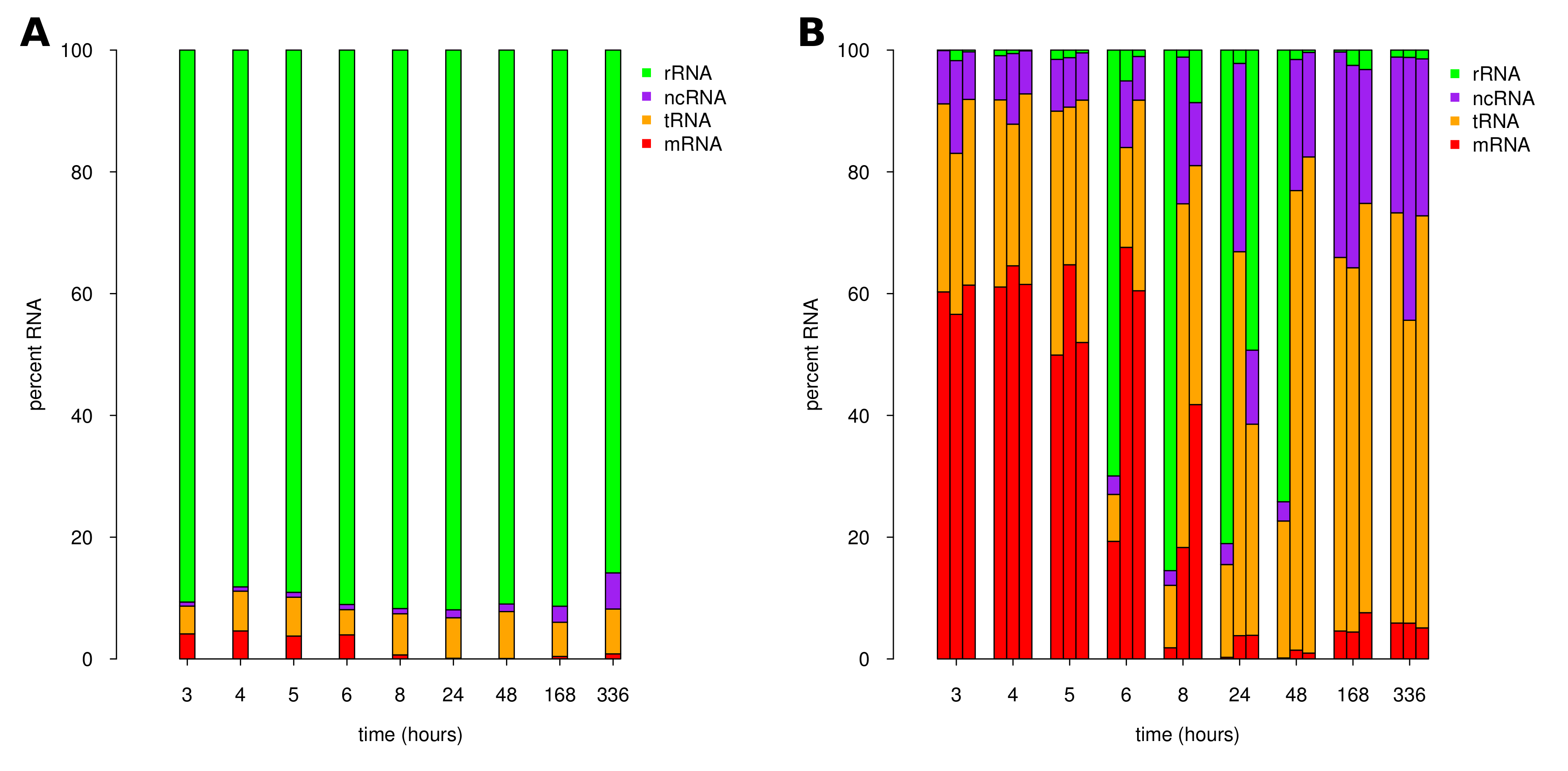

Supplement: S3 Fig — For each time point the fraction of total RNA reads in the RNA-seq results that mapped to tRNA (orange), rRNA (green), mRNA (red), or other noncoding RNA (purple) are shown. (A) RNA fractions for each total RNA sample that was processed without the rRNA depletion step. (B) RNA fractions for rRNA-depleted samples. Each bar represents an individual biological repeat. In some samples the rRNA depletion was not as successful as in others (e.g., biological replicate 1 at the 8 h time point). Any residual rRNA counts were disregarded before analyzing relative RNA expression levels. (TIFF) [file pcbi.1004400.s007.tiff]

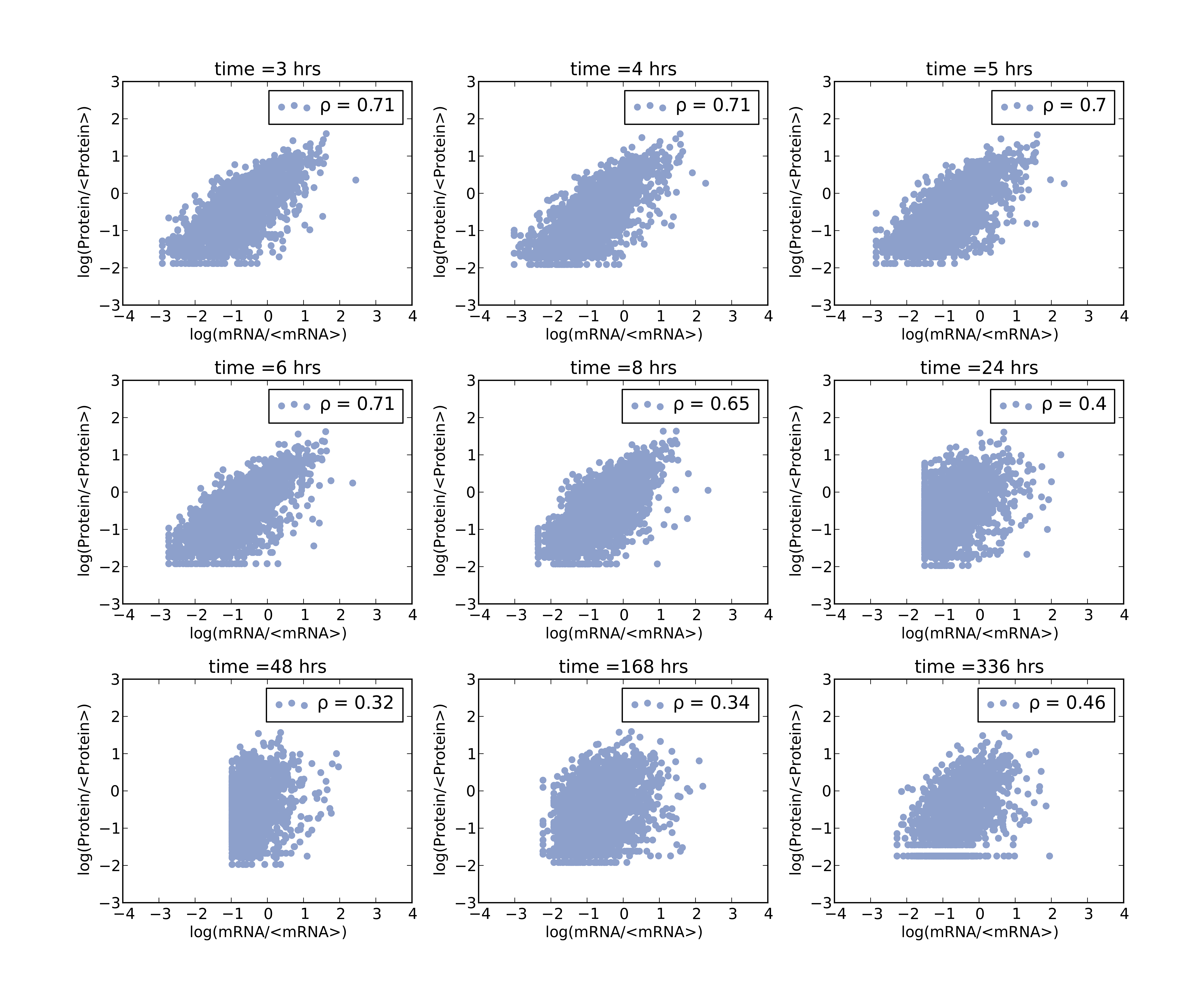

Supplement: S4 Fig — Both the RNA and protein levels were scaled to their respective averages across all RNAs or proteins for each time point and then log transformed. All P values were <10−43. (TIFF) [file pcbi.1004400.s008.tiff]

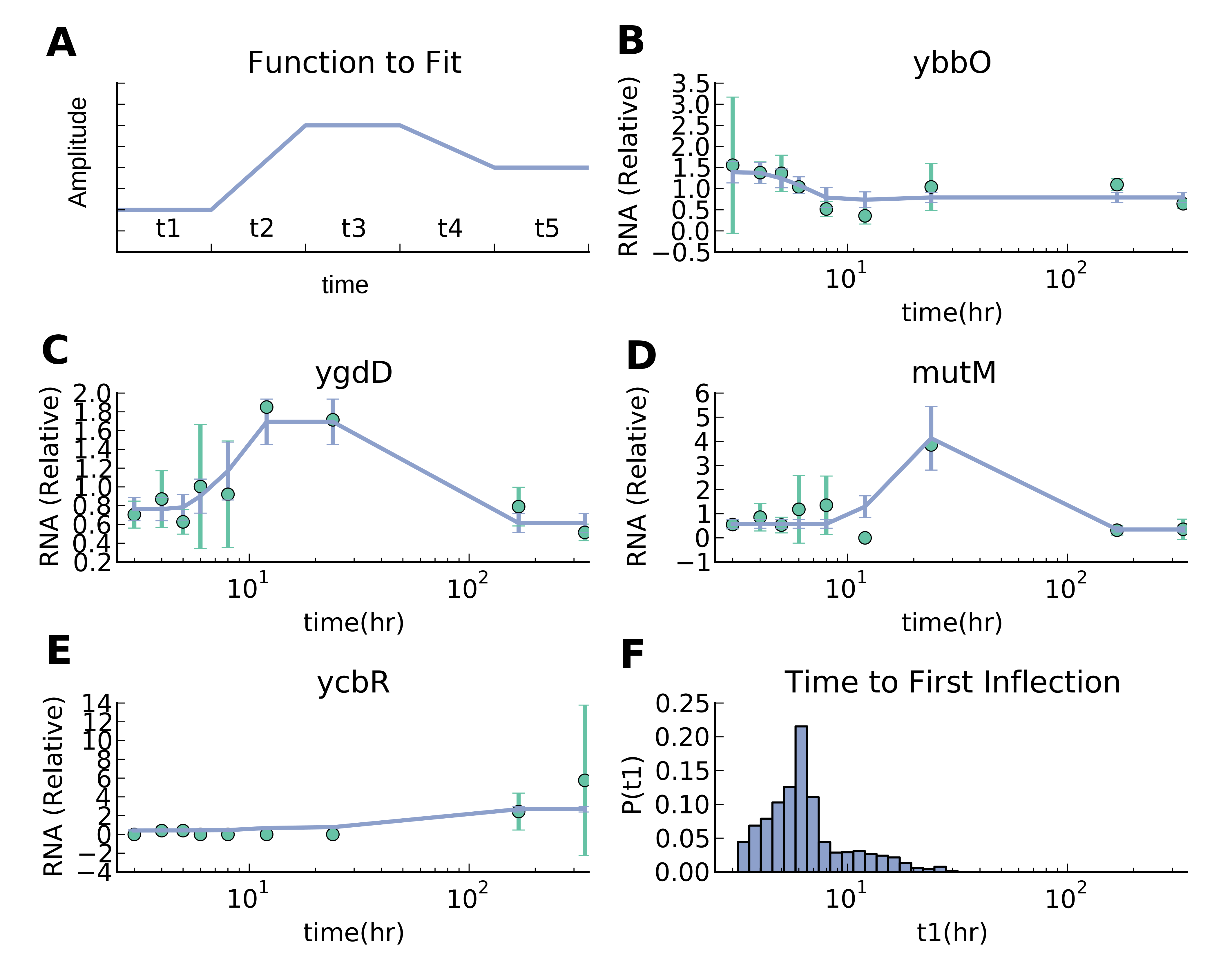

Supplement: S5 Fig — We grouped RNA and protein time courses based on general qualitative behaviors. After entry to stationary phase, RNA and/or protein can be shut off, turned on, transiently activated, or transiently repressed. (A) To sort the profiles, a piecewise continuous curve was fit to the data. The parameter t 0 represents the time at which we start to collect data at 3 h into growth. The curve was fit using a differential evolution fitting algorithm that was gradient free and population based, allowing for a range of possible parameter sets that can explain our data given the experimental error. (B-E) Four random examples of measured RNA time courses averaged across 3 biological replicates (green circles) with their standard deviations (green bars) along with the corresponding fits (blue). The blue bars represent the standard deviation of the range of fits that agree with our data. Both experimental time courses and fits were normalized by the average of the time course. (F) Most of the RNAs began to change between 6–8 h, when the cells began to be starved. This is demonstrated by the histogram of t 1, the time to the first inflection point. (TIFF) [file pcbi.1004400.s009.tiff]

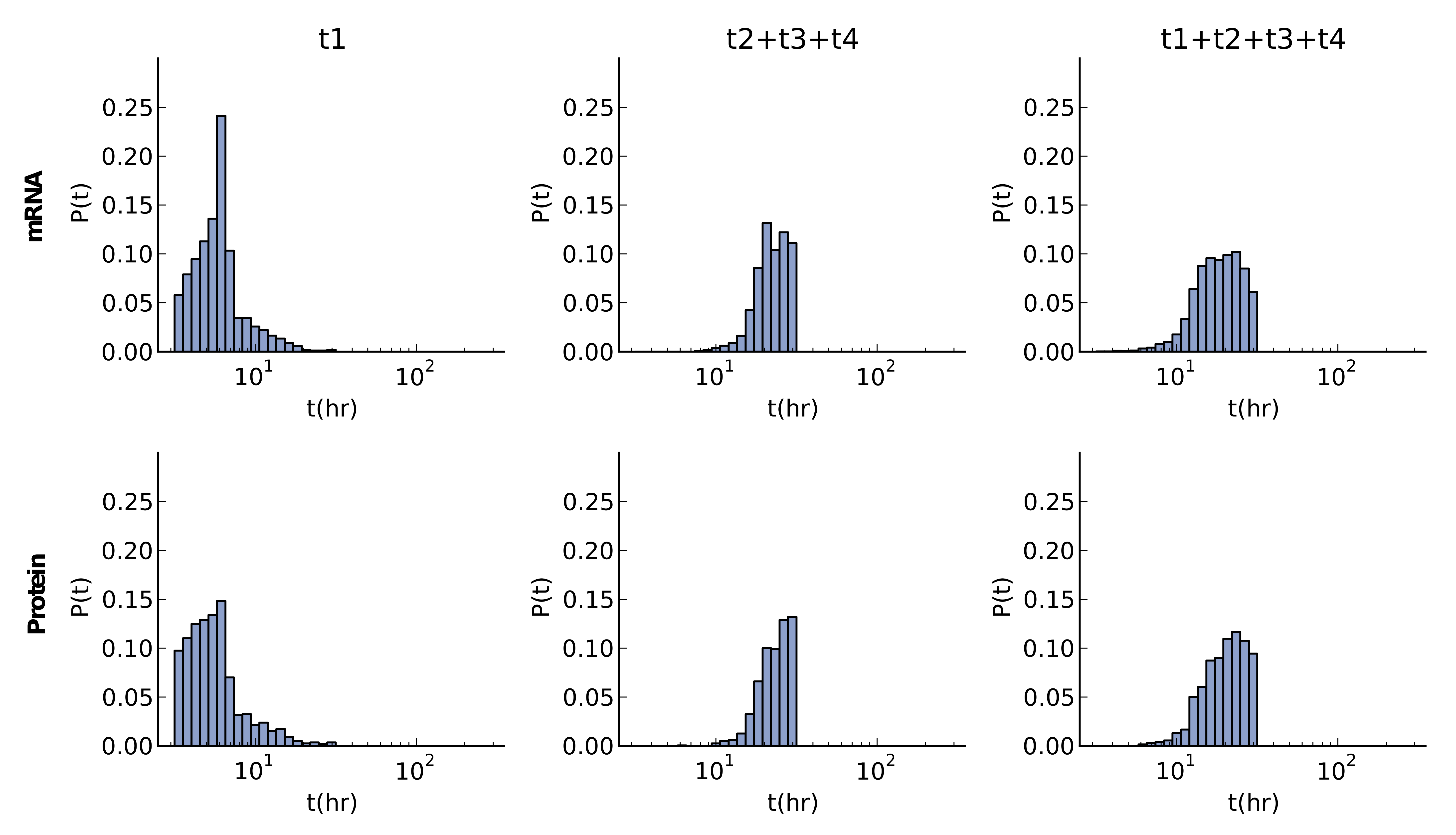

Supplement: S6 Fig — (A-C) mRNA distributions of t 1, time to first inflection (A), t 2 + t 3 + t 4, the time between the first inflection and time the profile levels off (B), and t 1 + t 2 + t 3 + t 4, the total time until a given profile levels off (C). (D-F) As (A-C), but for protein profiles. (TIFF) [file pcbi.1004400.s010.tiff]

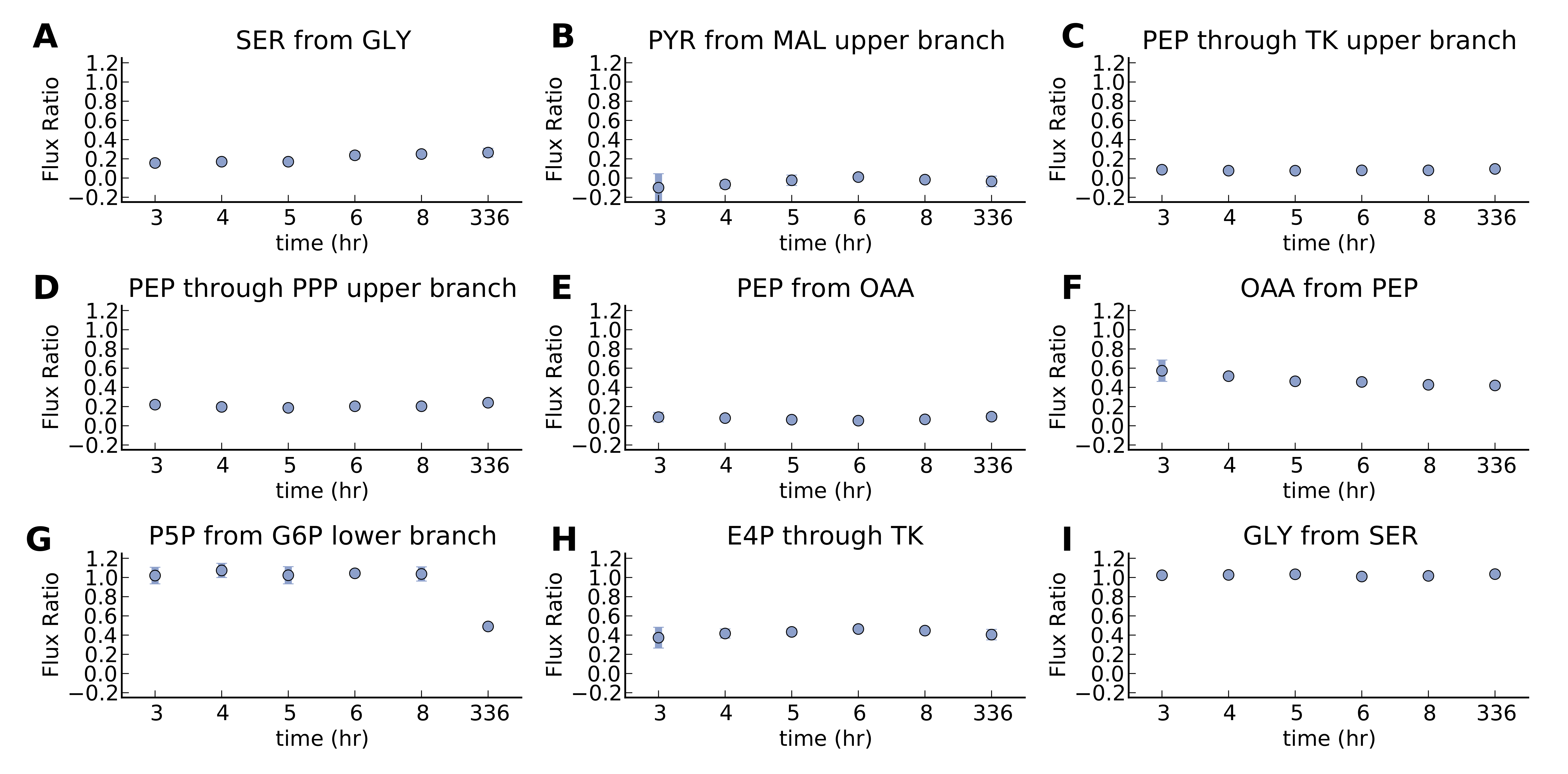

Supplement: S7 Fig — Flux ratios were computed via the FiatFlux software from GC-MS derived 13C constraints. As FiatFlux considers each time point as an integral from the start of the experiment, this analysis allowed us to determine whether later time points during growth changed the overall central metabolic flux splits that were estimated from earlier time points. Flux ratios for (A) SER from GLY, (B) OYR from MAL upper branch, (C) PEP through TK upper branch, (D) PEP through PPP upper branch, (E) PEP from OAA, (F) OAA from PEP, (G) P5P from G6P lower branch, (H) E4P through TK, and (I) GLY through serine. (TIFF) [file pcbi.1004400.s011.tiff]
